# Snake venoms are integrated systems, but abundant venom proteins evolve more rapidly

**DOI:** 10.1186/s12864-015-1832-6

**Published:** 2015-08-28

**Authors:** Steven D. Aird, Shikha Aggarwal, Alejandro Villar-Briones, Mandy Man-Ying Tin, Kouki Terada, Alexander S. Mikheyev

**Affiliations:** Okinawa Institute of Science and Technology Graduate University, Tancha 1919-1, Onna-son, Kunigami-gun, Okinawa-ken 904-0412 Japan; University School of Environment Management, Guru Gobind Singh Indraprastha University, Sector 16C, Dwarka, New Delhi 110078 India; Okinawa Prefectural Institute of Health and the Environment, Biology and Ecology Group, 2003 Ozato, Ozato, Nanjo-shi, Okinawa 901-1202 Japan; Research School of Biology, Australian National University, Canberra, ACT 0200 Australia

## Abstract

**Background:**

While many studies have shown that extracellular proteins evolve rapidly, how selection acts on them remains poorly understood. We used snake venoms to understand the interaction between ecology, expression level, and evolutionary rate in secreted protein systems. Venomous snakes employ well-integrated systems of proteins and organic constituents to immobilize prey. Venoms are generally optimized to subdue preferred prey more effectively than non-prey, and many venom protein families manifest positive selection and rapid gene family diversification. Although previous studies have illuminated how individual venom protein families evolve, how selection acts on venoms as integrated systems, is unknown.

**Results:**

Using next-generation transcriptome sequencing and mass spectrometry, we examined microevolution in two pitvipers, allopatrically separated for at least 1.6 million years, and their hybrids. Transcriptomes of parental species had generally similar compositions in regard to protein families, but for a given protein family, the homologs present and concentrations thereof sometimes differed dramatically. For instance, a phospholipase A_2_ transcript comprising 73.4 % of the *Protobothrops elegans* transcriptome, was barely present in the *P. flavoviridis* transcriptome (<0.05 %). Hybrids produced most proteins found in both parental venoms. Protein evolutionary rates were positively correlated with transcriptomic and proteomic abundances, and the most abundant proteins showed positive selection. This pattern holds with the addition of four other published crotaline transcriptomes, from two more genera, and also for the recently published king cobra genome, suggesting that rapid evolution of abundant proteins may be generally true for snake venoms. Looking more broadly at *Protobothrops*, we show that rapid evolution of the most abundant components is due to positive selection, suggesting an interplay between abundance and adaptation.

**Conclusions:**

Given log-scale differences in toxin abundance, which are likely correlated with biosynthetic costs, we hypothesize that as a result of natural selection, snakes optimize return on energetic investment by producing more of venom proteins that increase their fitness. Natural selection then acts on the additive genetic variance of these components, in proportion to their contributions to overall fitness. Adaptive evolution of venoms may occur most rapidly through changes in expression levels that alter fitness contributions, and thus the strength of selection acting on specific secretome components.

**Electronic supplementary material:**

The online version of this article (doi:10.1186/s12864-015-1832-6) contains supplementary material, which is available to authorized users.

## Background

Selection acts differently on different proteins, depending on their function. Understanding mechanisms underlying these differences has been a major thrust in molecular evolutionary biology [1–6]. A dominant pattern observed in studies of diverse model systems, ranging from yeast to mammals, is that secreted proteins evolve faster than intracellular proteins [7–11]. Reasons for this phenomenon remain poorly understood. Non-adaptive explanations posit that extracellular proteins experience relaxed selection, and are more tolerant of mutations for structural, ecological, or evolutionary reasons [8, 9]. Alternatively, extracellular proteins may play a larger role in evolutionary interactions with the environment and other organisms, which should make them more likely targets of positive selection [10, 11], as predicted by coevolutionary theory [12, 13].

In our view this uncertainty results from methodologies used in previous studies, which focused on large-scale analysis of evolutionary rate correlates. Although some authors could identify major genome-wide patterns, given the large number of genes involved, they could not determine proximal ecological factors that drive selection acting on extracellular gene products. Here we use snake venoms as a model system to illuminate the interplay between ecology and evolutionary rates of secreted proteins. Consisting of analytically tractable numbers of components with distinct pharmacological roles, snake venom proteomes have well-defined ecological roles in the immobilization of prey. Thus, comparing the composition of snake venoms between species as a function of prey choice and venom protein expression level, can provide general insights into how secreted proteins evolve.

Snake venoms function as integrated systems. Roles of individual constituents depend upon their concentrations and their interactions with other venom components [14]. Moreover, venom constituents also interact with compounds in prey tissues, most often in highly specific ways. Snake venom composition must respond to ontogenetic and evolutionary changes in diet, and presumably must also compensate to overcome predator or prey resistance, should that develop [15–17]. As a result, venoms from different species, and even those from different populations of the same species, appear optimally targeted to the chemistries of specific prey species. Furthermore, the structures of snake venom proteins evolve extremely rapidly (with a ratio of non-synonymous to synonymous substitutions greater than one), suggesting intense, positive, Darwinian selection. Consisting of mixtures of specific, evolutionarily conserved compounds with discrete pharmacologies, snake venoms provide an unusual opportunity to quantitatively study genomic consequences of ecological interactions, particularly how natural selection acts on rapidly co-evolving gene complexes.

Numerous and diverse studies attest to the strength of selection operating on venom composition. Various studies document a close match between venom composition and its effectiveness in subduing prey, suggestive of adaptation by the snakes. For example, Mackessy [18] showed that northern and southern Pacific rattlesnakes (*Crotalus viridis oreganus* and *C. v. helleri*) exhibit a pronounced shift in venom chemistry when young adults switch from lizard to rodent prey. The venom becomes less toxic, but much more proteolytic, presumably to enhance digestion of more voluminous prey with much smaller surface-to-volume ratios [19], although McCue [20] has disputed this conclusion. Daltry et al. [21] opined that venom variation in the Malayan pitviper (*Calloselasma rhodostoma*) is closely associated with diet. Chijiwa and colleagues [22] documented stark differences in venom phospholipases A_2_ (PLA_2_s) in populations of habus (*Protobothrops flavoviridis*) on different islands in the Ryukyu Archipelago. They inferred that these compositional differences reflect different selective pressures related to preferred prey. Experimental studies have shown that coral snake venoms are most toxic to preferred prey organisms [23]. Gibbs and Mackessy [24] found that toxicity of four pygmy rattlesnake (*Sistrurus*) venoms was correlated with the proportion of mice and lizards in the diet, with each taxon being most toxic to its preferred prey species. Conversely, the sea snake, *Aipysurus eydouxii*, feeds exclusively on fish eggs, obviating the necessity of venom. As a result, selection on its venom PLA_2_s has relaxed, leading to a range of dysfunctional mutations [25, 26].

In addition, many studies that focused on individual toxins or toxin classes have discovered evidence of accelerated protein sequence evolution, consistent with positive selection. It is well documented that contrary to normal tissue isozymes, venom protein exons diversify much more rapidly than introns [27–31]. In most venomous snakes, toxin gene duplication followed by neofunctionalization, has resulted in diverse pharmacologies [32, 33]. As an example, PLA_2_s manifest presynaptic neurotoxicity, myotoxicity, cardiotoxicity, anticoagulation, and cause vascular perforation (hemorrhage), hemolysis, and pain [34–36]. Although not as well studied as PLA_2_s, other snake toxin classes including cysteine-rich secretory proteins (CRISPs), disintegrins, C-type lectins, serine proteases, metalloproteases, and three-finger toxins all show evidence of rapid gene diversification, or of positive Darwinian selection [33, 37–43]. Moreover, the most rapidly evolving residues are those that appear on toxin surfaces, controlling their interactions with molecular targets in prey organisms [32, 44].

Some have suggested that accelerated evolution is predominantly a product of intrinsic mechanisms that enhance genetic diversity, independent of selective pressures. Kini and Chan [44] reasoned that accelerated evolution results from relaxed rather than directional selection. Mebs [45] argued that there is no evidence that strong selective forces drive the development of more potent toxins to overcome prey resistance, or to exploit new biochemical pathways for more effective intoxication of prey. More recently Kini and Chinnasamy [46] proposed that triplet nucleotide sequences determine the spontaneous mutation rate, an idea that requires further consideration.

In addition, phylogeny clearly plays a role in venom composition. More closely related species generally have more similar venoms than more distantly related species (e.g. the four mamba species have venoms that are qualitatively similar, but distinctly different from those of all other elapids). However, geography, and reduced gene flow may also influence venom chemistry. Angulo *et al.* [47] found pronounced differences in venom composition (only ~15 % similarity) between *Atropoides nummifer* and *Atropoides picadoi*. Since adults of both species are predominantly rodent predators, the differences appear to have a phylogenetic, rather than an ecological or geographic basis. However, the earlier work of Jiménez-Porras makes it clear that geographic separation of conspecific populations also affects venom composition in *A. nummifer* [48]. Angulo *et al.* did not indicate the geographic origins of their specimens, except to say that all were collected in Costa Rica.

Although toxin components interact to immobilize prey [14], historically, most studies of snake venom constituents have focused on specific toxin classes, examining long-term evolutionary trends in a variety of snake taxa. Consequently, although macroevolutionary patterns of individual venom proteins are somewhat understood, their regulation and microevolution remain almost entirely unknown [49]. However, if individual toxins (gene products) fluctuate in abundance and importance over short evolutionary time scales [50], evolutionary analysis of individual venom constituents may be misleading, as individual genes may experience different selective pressures from lineage to lineage. Furthermore, rates of venom protein evolution may depend on the relative abundance of various venom protein classes, and on their relative importance in immobilizing the prey.

Here we examine the interplay between component abundance and evolutionary rate, using two closely related pitviper species, the habu (*Protobothrops flavoviridis*) and the Sakishima habu (*Protobothrops elegans*). The former is native to Okinawa and nearby islands, and the latter to the Yaeyama Islands, located 400 km to the southwest in the Ryukyu Archipelago. The Sakishima habu is most closely related to the Taiwan habu (*P. mucrosquamatus*) [51], which is not surprising since it occupies the Ryukyu Islands closest to Taiwan. In 1976, however, human activities released approximately 100 Sakishima habus in the vicinity of Itoman, Okinawa [52–54]. The geological isolation of the Yaeyamas and Okinawa, is estimated at about 1.6 million years, which matches patterns of divergence of several island endemics [51, 55–57], though molecular estimates place divergence between these species at about 10 million years [58].

**Fig. 1:**
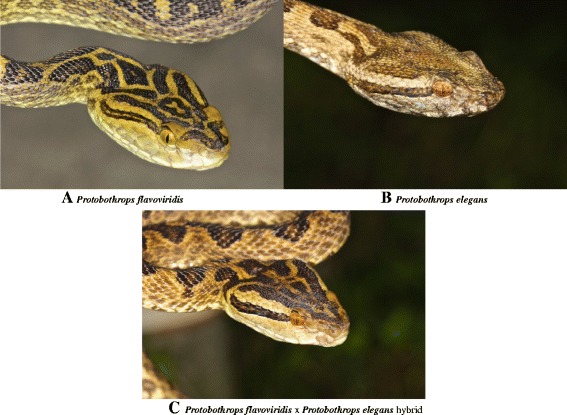
Photographs of the two parental species and a hybrid between them. **a** *Protobothrops flavoviridis*. **b** *P. flavoviridis* x *P. elegans* hybrid. **c** *Protobothrops elegans. Protobothrops flavoviridis*, as the name implies, has a yellow-green ground color, whereas *P. elegans* tends to be more orange to red, and head markings are quite different. *Protobothrops flavoviridis* is also much larger, reaching a maximum length of 2.5 m. The intermediate markings and color of the hybrids provided the impetus for an investigation of their venom chemistry

Between 1990 and 2000, more than 500 Sakishima habus were captured and brought to the Prefectural Institute of Health and Environment [53]. During this same period, five specimens of intermediate morphology were collected, suggesting possible hybridization. Recent collection of two more intermediate specimens prompted us to investigate whether these specimens are actual hybrids. Using a combination of transcriptomic and quantitative proteomic techniques [42], we identified venom components and determined their abundances in three specimens each of both parental species, and in the two putative hybrids, all collected in the same local area (Fig. 1). Because the putative hybrids are rare, and could not be sacrificed for venom gland extraction, we used only proteomic analysis of their venoms. We confirmed that they are genuine hybrids; however, serendipitously, these data also revealed some surprising patterns in the tempo and pattern of secreted protein evolution. Thus, an ostensibly simple question of possible pitviper hybridization unexpectedly illuminated fundamental evolutionary principles, which we could then verify in other species. Here then, are both intertwined stories.

## Results and discussion

We used a combination of transcriptomic and proteomic techniques to characterize the venoms of two closely related habus. By combining both approaches, we benefitted from the dynamic range and accuracy of transcriptomics, while proteomic data distinguished between proteins secreted into the venom, and non-venom components, allowing us to contrast evolutionary patterns in the two classes of genes. In addition, proteomics permitted us to quantify venom composition in rare and valuable hybrid specimens that could not be sacrificed for gland extraction. Unexpectedly, however, these hybrids provided insights into venom level regulation and evolution, showing heritability and additive regulation of venom component expression levels.

We also found a positive correlation between abundance of venom gene transcripts, and their evolutionary rates. This result is not obvious, given the non-stoichiometric action of enzymatic snake venom components, but it suggests that venomous snakes invest more in the production of proteins that are particularly important to them. Consequently, these proteins experience high rates of evolution, often with ω > 1, and show evidence of positive selection (Fig. 6; Additional file 1: Table S3). We propose that regulation of venom constituent levels may be a major short-term mechanism by which snakes adapt to new prey types, and may be a driver of protein molecular evolution.

The two *Protobothops* venoms were qualitatively similar at the level of protein families, and had many one-to-one gene homologs (Fig 3). Both venoms were heavily dominated by phospholipases A_2_, serine proteases, metalloproteases, and BPP-CNP, in that order (Table 1). *Protobothrops flavoviridis* venom possesses Factor IX/X inactivators that are absent in *P. elegans* venom. LAO transcripts were 6× more abundant in habu venom and VEGF transcripts were 3× as numerous (Table 1). Toxins having the primary function of hypotension or anti-coagulation, account for at least 21 % more of the *P. flavoviridis* transcriptome than the *P. elegans* transcriptome (Table 1). Presently, it is not possible to know whether any of the phospholipases A_2_ in either venom are anticoagulant; however, Zhao *et al.* [59] present strong evidence that catalytic activity is required for anticoagulant activity. This implies that the major *P. elegans* myotoxic PLA_2_ is not anticoagulant, while the two major PLA_2_s of *P. flavoviridis* venom may be. Taken together, it appears that the envenomation strategy of *P. flavoviridis* is focused much more heavily on provoking hypotension and distributing venom proteins throughout the prey, strategies that makes sense for mammalian prey, with much smaller surface-to-volume ratios than lizards, on which even adult *P. elegans* feed.

**Table 1:** The *Protobothrops elegans* and *P. flavoviridis* transcriptomes are overwhelmingly dominated by PLA_2_s; however, the major PLA_2_s in each are pharmacologically very different. *P. flavoviridis* venom appears to be much more anti-coagulant, with coagulation Factor IX/X-binding proteins, and higher percentages of serine proteases, LAO, C-type lectins, 5′-nucleotidase, and phosphodiesterase [14]

Transcript toxin class	*Protobothrops elegans*	*Protobothrops flavoviridis*
Phospholipase A_2_	77.1 %	55.5 %
Serine protease	10.4 %	11.8 %
Metalloprotease P-II	4.8 %	11.3 %
Metalloprotease P-III	3.2 %	6.0 %
BPP-CNP	1.4 %	2.6 %
Factor IX/X Activator A		2.4 %
Factor IX/X Activator B		1.2 %
LAO	0.5 %	3.1 %
VEGF	0.5 %	1.7 %
Phospholipase B	0.2 %	0.3 %
C-Type Lectin B	0.2 %	0.3 %
CRISP	0.1 %	2.0 %
5′-Nucleotidase	0.1 %	0.3 %
Nerve Growth Factor	0.1 %	0.1 %
Phosphodiesterase	0.1 %	0.2 %
Metalloprotease P-I	0.1 %	
C-Type Lectin A	0.0 %	0.6 %
Galactose-binding Lectin		0.0 %
QC	0.0 %	0.1 %
Hyaluronidase		0.0 %
DPP-IV	0.0 %	0.0 %
APA	0.0 %	0.0 %

Herein we address the composition of parental and hybrid venoms, confirmation of the hybrid status of the putative hybrids, and how natural selection acts on venoms as integrated systems.

### Composition of parental and hybrid venoms

#### Phospholipases A_2_

Both *Protobothrops* venom gland transcriptomes were dominated by phospholipases A_2_ (PLA_2_s). In the *Protobothrops elegans* transcriptome, two PLA_2_s comprised 76.9 % of all transcripts whereas in *P. flavoviridis* four isozymes represented 55.5 % (Table 1; Additional file 2: Table S1 and Additional file 3: Table S2). The PLA_2_ content in the *P. elegans* transcriptome is very high, but reasonable in light of pit viper venom chemistry. For example, in some neotropical rattlesnake (*Crotalus durissus*) venoms, crotoxin, a heterodimeric, PLA_2_ neurotoxin can comprise as much as 88 % of total venom protein [60–62].

**Fig. 2:**
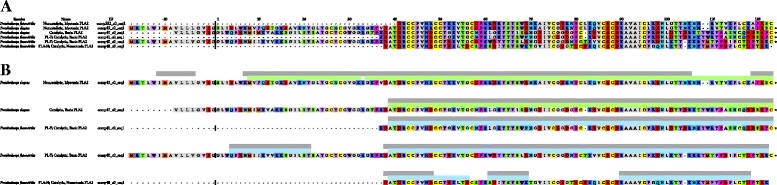
*Protobothrops flavoviridis* x *P. elegans* hybrids express phospholipases A_2_ from both parental venoms. **a** Aligned PLA_2_ transcripts from *P. flavoviridis* and *P. elegans* transcriptomes. The solid heavy vertical line separates the signal peptides from the expressed PLA_2_s. A noncatalytic, myotoxic PLA_2_ transcript (comp43_c0_seq1) from *P. elegans* specimen #3 accounted for 73.4 % of all *P. elegans* transcripts (Additional file 2: Table S1). This protein was heavily expressed in all three specimens and in both hybrids (Fig 3). A homologous transcript was found in the transcriptome of *P. flavoviridis* specimen #3, but it constituted less than 0.05 % of all *P. flavoviridis* transcripts (Additional file 3: Table S2). No peptides from this myotoxic PLA_2_ transcript were detected in venoms of any P. flavoviridis specimens, or of the hybrids (Fig 3). *P. elegans* transcript comp47_c0_seq1 is homologous to *P. flavoviridis* transcript comp41_c0_seq1 (Fig 3). *P. elegans* specimens expressed the PLA_2_ corresponding to the former transcript, while *P. flavoviridis* specimens expressed the latter. Hybrids expressed both (Fig 3; Additional file 8: Figure S5). The *P. flavoviridis* transcriptome also contained two additional PLA_2_ trancripts, comp40_c0_seq1 and comp48_c0_seq1 (Additional file 3: Table S2). These had no homologs in the *P. elegans* transcriptome, but hybrids produced both of these, as did all three *P. flavoviridis* specimens (Fig 3; Additional file 8: Figure S5). **b** Peptide coverage of venom PLA_2_ transcripts is similar between hybrids and non-hybrid specimens. Peptides from *P. elegans* venoms are indicated by green bars above the sequences, while those from *P. flavoviridis* venoms are in blue and those of hybrids are in gray. For any given transcript, peptides were sequenced from essentially the same portions of the PLA_2_ in both hybrid and non-hybrid venoms

**Fig. 3:**
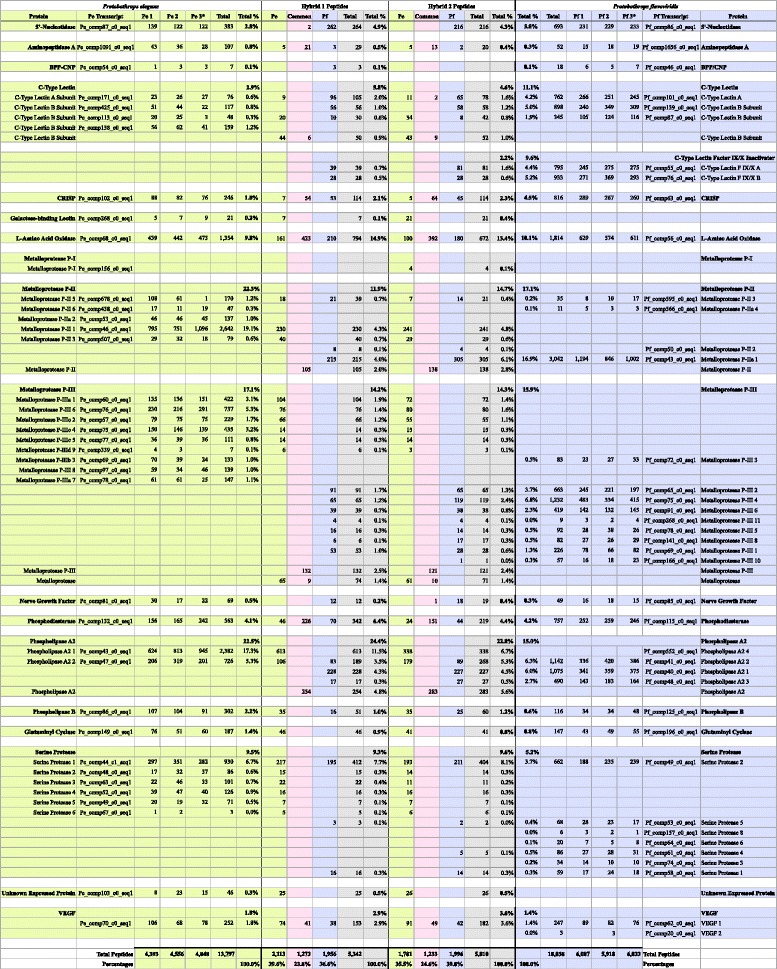
Proteomic data demonstrate that the two hybrids are genuine. Peptides from all specimens were matched to transcripts from both parental species (*specimens Pe 3 and Pf 3). Homologous proteins are aligned on the same row. Proteins without homologs in the other transcriptome occupy their own rows. Percentages at the bottom of the hybrid columns indicate the percentage of all peptides in the hybrid proteome corresponding to transcripts in each of the parental species. For the hybrids, numbers of unique peptides corresponding to each of the parental transcripts are provided in color-coded columns (green = P. elegans; blue = P. flavoviridis). If a peptide could have come from one or more transcripts in both parental species, it was classified as “common” (pink), meaning that it was potentially common to both, or that its origin was indeterminate. Total percentages of peptides from each protein family are given in bold. Hybrid percentages for each pair of venom protein homologs are shown in gray columns. Peptides that corresponded to missing portions of incomplete transcripts could not be identified. Despite huge advances in mass spectrometric technology, shotgun sequencing is still less quantitative than transcriptomic approaches, so proteomic totals differ quantitatively from those presented for transcriptomes (Additional file 2: Table S1 and Additional file 3: Table S2) In the Phospholipase A2 section, 613 peptides from a noncatalytic, myotoxic PLA2 (transcript Pe_comp43) were detected in the venom of Hybrid 1 and 338 peptides were isolated in venom of Hybrid 2. It was also found in venoms of all three P. elegans specimens. Neither the hybrid venoms nor the three P. flavoviridis venoms contained peptides from the homologous PLA2 (transcript Pf_comp552). Likewise, peptides from two P. flavoviridis PLA2 transcripts (Pf_comp40 and 48), with no homologs in the P. elegans transcriptome, were found in the venoms of all three P. flavoviridis and those of both hybrids, but not in the three P. elegans venoms. In contrast, P. elegans transcript Pe_comp47 and P. flavoviridis transcript Pf_comp41 encode a basic, catalytic PLA2, homologous to PL-Y. Both hybrids had peptides corresponding to each of these transcripts while specimens of the two species had only their own Hybrid 1 had 254 common PLA2 peptides (pink) and Hybrid 2 had 283, comprising 4.8 and 5.6 % of the two hybrid proteomes, respectively. These peptides could have originated with transcripts in both parental venom proteomes and could not be assigned unambiguously to any single transcript. In Hybrid 1 venom, 24.4 % of all peptides detected were PLA2 peptides, while this class accounted for 22.8 % of the peptides identified in Hybrid 2 venom. Most other protein families tell a similar story. Venom composition of the putative hybrid venoms confirm that the specimens are legitimate hybrids

Based on homology to other PLA_2_s of known pharmacology, *P. elegans* transcript comp43_c0_seq1 that constituted 73.4 % of all transcripts, encodes a noncatalytic, myotoxic PLA_2_ myotoxin that shows considerable homology to other Asian crotaline myotoxins (Fig. 2a; Additional file 4: Figure S1A; Additional file 5: Figure S3; Additional file 2: Table S1). In contrast, while *P. flavoviridis* also has a transcript (Pf_comp552_c0_seq1) for a homologous, non-catalytic, myotoxic PLA_2_, this transcript represents <0.05 % of the latter transcriptome. Both the *P. elegans* and *P. flavoviridis* myotoxins have an arginine residue (position 48) where catalytic PLA_2_s have aspartic acid in order to bind the Ca^2+^ ion required for catalysis. Many New World crotalines have lysine in this position. All three *P. elegans* specimens and the two hybrids express Pe_comp43_c0_seq1 heavily, but no snakes in this study produced the *P. flavoviridis* homolog, Pf comp552_c0_seq1 (Fig 3; Fig. 2; Additional file 6: Figure S5).

All other PLA_2_s in both venoms are presumably catalytic. The dominant PLA_2_s in *P. flavoviridis* venom (Pf comp41_c0_seq1 and comp40_c0_seq1) are both basic, as is *P. elegans* comp47_c0_seq1, which is homologous to Pf comp41_c0_seq1. Their pharmacologies are unknown. All three *P. elegans* venoms contained Pe_comp47_c0_seq1, but none of the *P. flavoviridis* PLA_2_s. Likewise, all three *P. flavoviridis* venoms contained the three Pf PLA_2_s, but not Pe comp47_c0_seq1. The hybrids, however, produced all five PLA_2_s from both parental species (Fig 3; Fig. 2; Additional file 6: Figure S5).

*Protobothrops elegans* venom apparently has no neurotoxic PLA_2_s, but *P. flavoviridis* comp48_c0_seq1, which encodes a weak presynaptic neurotoxin similar to trimucrotoxin [63], comprised 6.3 % of the *P. flavoviridis* transcriptome. Although some neurotoxic PLA_2_s are also myotoxic [64, 65], myotoxicity appears to be much more important for *P. elegans* than for *P. flavoviridis*. Nonetheless, it is difficult to offer an ecological explanation for this difference. In general, myotoxicity seems to be most often associated with mammal predation by large terrestrial crotalines [66–68], but it has also been reported in small arboreal species [69]. Without appropriate pharmacological studies in native prey species as well as laboratory animals, it is impossible to know whether such differences are adaptive or ecologically irrelevant. It may be that some subsets of myotoxic PLA_2_s are specifically adapted to reptilian rather than mammalian skeletal muscle, but if so, the structural determinants are currently unknown.

Relative to PLA_2_s, one other matter deserves mention. The *P. elegans* cDNA library contains a peculiar transcript, Pe_comp103_c0_seq1, that represented 0.2 % of the transcriptome (Additional file 2: Table S1). Using the Frame 3 reverse translation of comp103_c0_seq1, BLASTP and TBLASTX searches both suggested that the best match is a PLA_2_ gene cluster from *P. flavoviridis* (in Frame 1) [70]. However, both sequences are liberally punctuated with stop codons, and the longest, unbroken, translated segment of the *P. elegans* transcript, using Frame 1, encodes only 45 amino acids, or about 37 % of a typical Type II PLA_2_. BLASTP and pfam searches for the 45-residue protein yielded no results. However, mass spectrometry of the eight venoms detected three peptides (a total of 46 times) that cover 55.8 % of the putative protein translated in Frame 1 (Fig 3). Further analysis of these peptides was done using de-novo sequencing algorithms and manual annotation, in order to confirm their identity (Additional file 7: Figure S2). This protein was not only detected in all three specimens of *P. elegans*; it was also detected in both hybrid venoms (Fig 3). Clearly, the snakes are producing this, but it does not appear to be a PLA_2_ derivative, the TBLASTX searches notwithstanding. Its function, assuming that it has one, is unknown.

#### Serine proteases

In both transcriptomes, serine proteases (SPs) are the second most abundant protein family (Table 1). Six SPs comprise 10.4 % of all *P. elegans* transcripts, whereas eight represent 11.8 % of all *P. flavoviridis* transcripts. Based on homology, *P. elegans* SP 1 (5.5 % of all transcripts) (Additional file 2: Table S1), is a thrombin-like enzyme (TLE) and a possible kallikrein. *Protobothrops elegans* SPs 2, 3, and 6 are probable TLEs, while based on BLASTP searches, SPs 4 and 5 are probable plasminogen activators. *Protobothrops flavoviridis* SPs 1 and 4 are probably TLEs, based on sequence homology to DAV-PA from *Deinagkistrodon acutus* venom (Additional file 3: Table S2). *Protobothrops flavoviridis* SP2, a complete transcript, is an isoform of a serine protease variously known as habutobin or flavoxobin. It is a strongly anticoagulant thrombin-like enzyme that releases only fibrinopeptide A [71, 72], inhibits collagen-induced platelet aggregation [73], and releases urokinase-tissue plasminogen activator from bovine arterial endothelial cells [74]. In addition, it functions as a C3-convertase that activates the complement cascade [75]. SP5 most closely resembles KN5 from *Viridovipera stejnegeri* venom (Tsai and Wang, direct submission: http://www.ncbi.nlm.nih.gov/protein/82239734), for which no pharmacological data are available. SP7 is a probable plasminogen activator, based on similarity to another homolog from *V. stejnegeri* venom [76]. The majority of the SPs in both venoms appear to function in multiple ways as anticoagulants. As with PLA_2_s, the hybrids produce SPs from both parental species, but all peptides could be attributed to specific parental transcripts; there were no peptides of ambiguous (potentially common) origin.

At first glance, it might appear contradictory for snakes to employ both procoagulant and anticoagulant enzymes, but in reality, this is a well-coordinated strategy to render the prey’s blood wholly incoagulable with astonishing speed. Venom thrombin-like enzymes (TLEs) consume fibrinogen, yielding small fibrin clots, and hypotensive fibrinopeptides [77, 78]. However, the commencement of inappropriate clotting triggers the prey’s anti-clotting cascade, which rapidly destroys the fibrin clots [79]. *Protobothrops flavoviridis* venom contains a Factor IX/X inactivator (3.6 % of the transcriptome) that is lacking in *P. elegans* venom, and kallikrein-like enzymes, which *P. elegans* does have (Additional file 2: Table S1, Additional file 3: Table S2). But kallikrein converts plasminogen to plasmin, which in turn, digests fibrin. Various venom serine proteases have both thrombin-like and kallikrein activities [80–85]; thus fibrinogen-clotting and plasminogen activation reside in the same protein. Regardless, many crotaline venoms also contain serine and metalloproteases that degrade fibrin directly [86, 87].

Many snake venom TLEs clot fibrinogen less effectively than thrombin [88] and TLEs are commonly more effective against fibrinogens of some mammal species than others [89], but the existence of so many weakly clotting TLEs, the capacity of various crotaline TLEs to degrade prothrombin [90], and the existence of directly fibrinolytic venom enzymes, confirm that the objective is not to clot blood, but to clear the bloodstream of fibrinogen [91]. Even in human envenomations by small crotalines, blood can be rendered incoagulable within minutes [92]. Defibrinogenation would occur in small mammalian prey far more rapidly. Incoagulable blood presumably facilitates the distribution of venom and endogenous prey tissue hydrolases, promoting prey digestion. In addition to fibrin cleavage, plasmin also inactivates many endogenous clotting factors, thereby acting as an anticoagulant [93]; however, this also suggests that the strategy may be to prevent endogenous coagulation factors from producing properly clotted fibrin. For a more detailed look at the role of serine proteases in envenomation, see the Additional file 9.

#### Metalloproteases

In both transcriptomes, P-II metalloproteases are the third most abundant protein family (*P. elegans*: 4.8 % and *P. flavoviridis*: 11.3 %), followed by P-III metalloproteases (*P. elegans*: 3.2 % and *P. flavoviridis*: 6.0 %) (Table 1). *Protobothrops elegans* venom contains as many as six P-II MPs, one apparent P-I MP, and as many as nine P-III MPs. However, caution must be exercised in predicting the number of members of these highly diversified families. From previous experience, it is clear that partial transcripts of large proteins tend to overestimate numbers of homologs and paralogs in these families [42]. Our earlier study suggested that there could be as many as 12 P-II MPs and as many as 9 P-III MPs in *P. flavoviridis* venom. The present study found evidence for no more than 4 P-II MPs, but suggested as many as 11 P-III MPs (Additional file 3: Table S2). The problem seems to be that the Trinity assembler is unable to deal effectively highly diversified families of large proteins, such as MPs and SPs. Reassembly of both transcriptomes using the new assembler, VTBuilder, suggested assembling two pairs of these (Pf_comp65_co_seq1 with Pf_comp75_c0_seq1; Pf_comp69_c0_seq1 with Pf_comp72_c0_seq1), resulting in an estimated 7 P-III MPs. Nonetheless, overall, the two studies present a unified view of the relative importance of the various toxin families.

The putative P-I metalloprotease transcript identified in the *P. elegans* transcriptome had no apparent homolog in *P. flavoviridis*. Oddly, while peptides from this protein were not identified in any of the *P. elegans* venom samples, Hybrid #2 did express it. P-II metalloproteases were expressed in the hybrids at the slightly lower levels than those seen in either of the parental venoms. Four of five *P. elegans* enzymes were identified in the hybrids and three out of four from *P. flavoviridis*. In the two parental species, P-II metalloproteases were expressed somewhat more highly than P-III enzymes. MPs were expressed at about 12–15 % in both hybrids (Fig 3; Additional file 6: Figure S5).

It is difficult to predict metalloprotease pharmacology based upon subclass identity alone, owing to a dearth of comparative structure-function studies. The structural complexity of P-III enzymes in particular, gives rise to a great variety of pharmacologies, including hemorrhage, inflammation, apoptosis, fibrinogen and fibrin degradation, prothrombin activation, and platelet aggregation inhibition [94]. The extremely hemorrhagic nature of *P. flavoviridis* venom is well known [95–98], and that characteristic reflects the well-documented presence of hemorrhagic MPs [99–102]. P-III MPs 2, 4, 7, 9, and 11 are clearly isomers of HR1, a hemorrhagic P-III MP (Additional file 3: Table S2) [103–106]. MP P-IIIs 1, 3, 5, 6, 8, and 10 appear homologous to flavorase (HV1), characterized as an apoptosis-inducing protein (Additional file 3: Table S2) [107]; however, apoptosis is too slow a process to be relevant to envenomation; its more important contribution to envenomation is probably to degrade fibrinogen [108].

Despite the presence of an apparent P-I MP in the *P. elegans* transcriptome, we are unable to say whether an independent P-I MP exists in this venom, or whether this represents an incomplete P-II or P-III transcript. The putative P-I transcript was itself incomplete and represented only 0.1 % of the transcriptome. Moreover, no peptides from this transcript were found in any of the three *P. elegans* venoms, although they were found in the venom of hybrid #2. Graminelysin I, from the venom of *Trimeresurus gramineus*, a P-I MP, has been shown to be processed post-translationally from a P-III precursor [109], but that situation is probably not analogous to this one.

#### Bradykinin-potentiating peptides and C-type natriuretic peptide

The gene that encodes multiple bradykinin-potentiating peptides and a single C-type natriuretic peptide (BPP-CNP) was found in both transcriptomes (*P. elegans*: 1.4 % and *P. flavoviridis*: 2.6 %) (Table 1). All of these gene products are hypotensive, at least in mammals, and cause prey to go into circulatory shock, limiting prey flight [14]. These peptides were more highly expressed in *P. flavoviridis* venoms and peptides from hybrid venoms corresponded only to the *P. flavoviridis* transcript (Fig 3; Additional file 6: Figure S5).

#### Other venom constituents

C-type lectins that act upon blood platelets were found in the transcriptomes and proteomes of both species and hybrid venoms showed peptides from both parental venoms (Fig 3; Additional file 6: Figure S5). *Protobothrops flavoviridis* produces an anticoagulant C-type lectin that binds and inactivates coagulation Factors IX/IXa and X/Xa [110, 111], but *P. elegans* does not. Both hybrids manifested this protein, but at levels substantially lower than seen in *P. flavoviridis* (Fig 3; Additional file 6: Figure S5).

L-amino acid oxidase represented 3.1 % of the *P. flavoviridis* transcriptome, but only 0.5 % in *P. elegans* (Tables 1, Additional file 2: Table S1 and Additional file 3: Table S2). However, LAO peptides accounted for 14.9 % of the *P. elegans* venom proteome and 13.4 % in *P. flavoviridis* (Fig 3). Vascular endothelial growth factor, was an even more minor element of the two transcriptomes (1.7 % of *P. flavoviridis*, but only 0.5 % of *P. elegans*). All other components represented less than 1 % of one or both transcriptomes (Tables 1, Additional file 2: Table S1 and Additional file 3: Table S2). These included phospholipase B, cysteine-rich secretory proteins, 5′-nucleotidase, nerve growth factor, phosphodiesterase, galactose-binding lectin, glutaminyl cyclase, hyaluronidase, dipeptidyl peptidase IV, and aminopeptidase A. Pharmacologies of these proteins relative to their roles in envenomation have been reviewed in Aird [14] and Aird et al. [42].

#### “Common” peptides in the hybrid proteomes

For most protein families, it was possible to detect in the hybrid venoms, a large number of unique peptides that could be assigned to specific transcripts from each of the parental species (Fig 3; Additional file 6: Figure S5). However, for some families there were a large number of peptides that could be matched to one or more transcripts in both parental venoms. That is, they were potentially common to both. Not surprisingly, the percentage of common peptides in the hybrid proteomes was greatest in the least diversified protein families. These families generally have only one transcript per species. Examples include APA (69.4 % common), LAO (55.6 %), PDE (67.2 %), and CRISP (51.8 %) (Fig 3). Two stark exceptions were 5′-nucleotidase and PLB (0 %). Highly diversified protein families showed much lower percentages of common peptides, the extreme case being serine proteases, for which no common peptides were found.

#### Quantitative proteomics of the hybrids

**Fig. 4:**
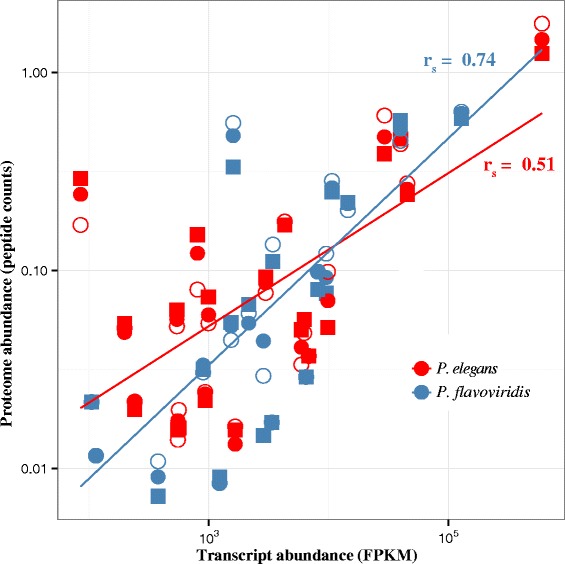
Transcriptomic and proteomic data present generally concordant pictures. When mapped against their own references, the proteomic and transcriptomic measures of abundance were correlated (Spearman rank *p* = 1.5 × 10^−9^, 5.3 × 10^−9^, for *P. elegans* and *P. flavoviridis*, respectively). Individual proteomic samples are represented by different shapes, with the sample used for the reference transcriptome shown as an open circle. Since only one transcriptome was sequenced for each species, biological replicates share the same X-coordinate. The methodology employed herein was that reported in [42]

Proteomic data can be quantified by standardizing absolute peptide abundance by the predicted protein length [42]. This approach produced significant correlations between protein and transcriptomic abundance estimates, with close agreement between protein sample replicates from different individuals (Fig. 4). However, reference transcripts were only available for the parent species, and not for the putative hybrids. In order to make comparisons of protein abundance, a common reference was needed. We identified peptides using each transcriptome as a reference, in order to determine which performed better. The *P. flavoviridis* reference produced the most significant correlation between transcriptomic and proteomic data for both species (Spearman correlation with *P. elegans*: r = 0.29, *p* = 0.011, *P. flavoviridis* r = 0.50, *p* = 5.3 × 10^−9^), so it was used to call peptides for the hybrids (Fig. 4).

**Fig. 5:**
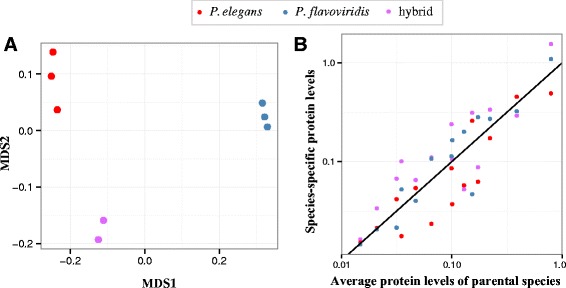
Hybrid snakes have protein expression profiles distinct from those of the parental species. **a** The non-metric multidimensional scaling (NMDS) analysis of protein composition and abundance showed that protein levels in the hybrid venom proteomes showed intermediate along the primary MDS axis. This analysis shows that the parental species and the hybrids have consistent protein expression levels. **b** Normalized counts of peptides detected by mass spectrometry for homologous proteins in the hybrids, compared to parental values, with y = x line plotted for reference. Although most of the hybrid data points lie outside the parental means (12 of 14, 1-sample proportions test, *p* = 0.00011), suggesting either under- or overdominance, common in hybrids, the overall the slope and the intercept of the best fit are not different from y = x (*p* = 0.36). Both analyses show that the hybrid proteomes are on average intermediate relative to those of *P. elegans* and *P. flavoviridis*, suggesting strong genetic effects on both their compositions and component levels

Non-metric, multidimensional scaling analysis of the putative hybrid proteomes showed that they were largely intermediate between those of the parental species (Fig. 5a). The expression level of homologous venom components was similarly intermediate (Fig. 5b).

### Confirmation of the putative hybrids

The two putative hybrids expressed all venom proteins from both parental species, except for those corresponding to the most minor transcripts (Fig 3; Additional file 6: Figure S5), and had distinct venom profiles (Fig. 5). Venom proteins produced by only one of the parental species also appeared in hybrid venoms (e.g., *P. elegans* PLA_2_ Pe_comp43_c0_seq1 and *P. flavoviridis* Factor IX/X inactivators, Pf_comp55_c0_seq1 and Pf_comp76_c0_seq1) (Fig 3; Additional file 5: Figure S3). They also had roughly intermediate levels of protein expression for most venom proteins, compared to the parental species (Fig 3, Fig. 5; Additional file 8: Figure S4; Additional file 6: Figure S5). The strong correlation between protein levels in the two parental species and in the hybrids indicates that venom levels are genetically controlled (Fig. 5). Hybrid venom protein levels are largely intermediate between those of the parental species, suggesting that each component may be largely additively regulated, though there is also evidence that hybrids show higher levels of proteins, relative to the two parental species, perhaps a manifestation of “hybrid vigor”. Additive effects, as opposed to dominance and epistatic effects, are particularly good targets for natural selection [112], though, more generally, genetic control of venom constituent levels makes them a potential target for natural selection. Consequently, rapid population-level changes in venom chemistry are expected in the face of sufficient selective pressure. This mode of regulation is consistent with the rapid evolution of snake venoms seen in *Protobothrops* and other taxa.

Given their morphological intermediacy, and the additive compositions of their venoms, it is apparent that the putative hybrids are, in fact, bona fide hybrids. We know of no other way to explain the “hybrid” nature of these venoms except for hybridization between the native *P. flavoviridis* and the invasive *P. elegans*.

While the morphologically intermediate animals (Fig. 1) are legitimate hybrids, without tissue samples, it is impossible to determine which parent pertained to which species, and we also cannot be certain whether the hybrids are F1 or F2 hybrids. Given the substantial intermediacy of hybrid venom composition relative to the two parental species, it is virtually certain that they do not represent back-crosses with either parental species, which would be expected to skew venom composition toward that parental species. Nonetheless, it is clear that *P. elegans* and *P. flavoviridis* are not reproductively isolated by pre-mating isolating mechanisms. Hybridization with invasive species can threaten conservation of *P. flavoviridis*, and future studies should address its extent and impact.

### How natural selection operates on venoms

#### Evolutionary rate analysis

Venom gland transcripts for which venom peptides were sequenced by mass spectrometry, had much higher rates of non-synonymous to synonymous (dN/dS) substitutions relative to tissue components of the venom gland transcriptomes (Kruskal-Wallis *p*-value = 1.8 × 10^−10^) (Fig. 5a). Among the proteins detected in either *P. elegans* or *P. flavoviridis* venom by mass spectrometry, there was a positive correlation between the average abundance of a toxin in the transcriptome, and its evolutionary rate (r = 0.53, d.f. = 24, *p* = 0.0055; Fig. 5b). Including only proteins detected in both venoms, the correlation remained, at lower significance, due to the decreased sample size (r = 0.48, d.f. = 17, *p* = 0.037). Protein abundance levels were likewise correlated with evolutionary rate (r = 0.62, d.f. = 17, *p* = 0.0041). By contrast, the transcriptional abundance of non-venom (tissue protein) transcripts was negatively correlated with evolutionary rate (r_s_ = -0.087, *n* = 1012, *p* = 0.0057). There was evidence of positive, significant selection acting on most venom proteins with dn/ds >1. PLA_2_s, metalloproteases and C-type lectins all showed evidence of positive selection in *Protobothrops* as a whole (*p* < 0.05). PARRIS failed to detect positive selection acting on serine proteases (*p* = 0.34, despite inferring dn/ds = 1.27; Table S3), but MEME found significant evidence of episodic diversifying selection at four sites, a result consistent with the absence of common peptides (Fig 3).

#### Control of expression levels

Differences in abundance of homologous proteins between *P. flavoviridis* and *P. elegans* are dramatic. It is possible that the expression level of each venom component is simply a function of its promoter strength. In such a case, we would predict that individual toxin levels should be weakly correlated with levels of other transcripts, and that promoter regions should show classical signs of positive selection, such as reduced haplotypic diversity, and strong sequence divergence between species. Both of these predictions can be tested by conducting a gene co-expression level analysis study, and an investigation of genetic diversity upstream of venom toxin genes, respectively.

#### Energetics and selection

**Fig. 6:**
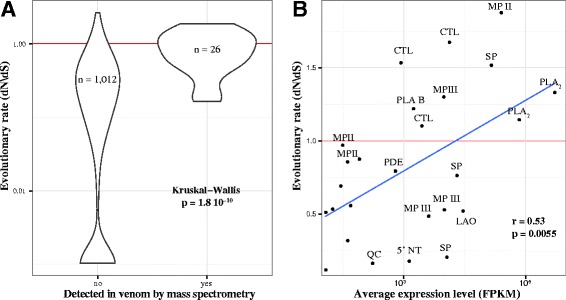
Secreted proteins evolve rapidly, and more abundant venom toxins evolve most rapidly of all. **a** Violin plot of evolutionary rates of secreted proteins (those detected by mass spectrometry in the venom) vs. the rest of the transcriptome. Secreted proteins evolve significantly faster than the rest of the transcriptome, suggesting that they are subject to atypically strong selection within the genome. **b** Relationship between abundance and nucleic acid evolutionary rate for secreted proteins. More abundant venom components, which likely represent a greater metabolic investment for the snake, but are also more likely to contribute to the venom’s effectiveness, evolve faster. Abbreviations: 5*′NT* 5′-nucleotidase, *CTL* C-type lectin, *LAO* L-amino acid oxidase, *MP* metalloprotease, *PDE* phosphodiesterase, *PLA* phospholipase, *SP* serine protease, *QC* glutaminyl-peptide cyclotransferase. Several unlabeled points represent proteins present in the venom at low concentrations, but without clear pharmacological significance

Although enzymatic venom constituents do not act stoichiometrically as non-catalytic toxins do, nonetheless, greater abundance necessarily signifies a greater role for the constituent in question in prey immobilization and/or digestion, regardless of the presence or absence of catalytic activity. Snake venoms are the most concentrated glandular secretions in the animal kingdom, with solutes, 90 % of which are proteins, comprising 25–35 % by mass [113, 114]. Given the high cost of venom production as a function of overall metabolism [114], more abundant components must necessarily carry a higher biosynthetic cost to the snake. Therefore, snakes should optimize the abundance of venom constituents to maximize immobilizing power, while minimizing cost. Both venom gene expression levels and gene product sequences should be under strong selective pressure. This seems to be the case generally in *Protobothrops*, at least for sequences above dN/dS = 1 (Fig. 6), which all showed some evidence of positive selection, in our study and in earlier studies of accelerated evolution, which covered C-type lectins, serine proteases and PLA_2_s [28, 30, 37, 115], and also in our re-analysis (Table S3). This suggests that the higher rates of evolution in more abundant components are due to adaptation, rather than to relaxed selection.

#### Venom proteins and tissue proteins

Comparison of two closely related species that became geographically isolated several million years ago [51, 57, 58], allowed us to examine the interplay between venom composition and evolutionary rate of venom proteins. Their venoms show much greater sequence divergence than venom gland cellular (tissue) components. Interestingly, while secreted venom components have evolved much faster than the cellular (non-secreted) portion of the venom gland transcriptome, more abundant venom proteins evolved most rapidly (Fig. 6). This pattern contrasted with the cellular portion of the venom gland transcriptome, where there was a negative relationship between protein abundance and evolutionary rate, similar to that found in most organisms [116, 117]. Together with data including other *Protobothrops* species, which show evidence for positive selection on the most abundant venom components (Table S3), this strongly argues against the suggestion that snakes inject so much venom into their prey that the resulting ‘overkill’ greatly relaxes selection on venom components [45, 118].

#### Extending the present analysis to other datasets

**Fig. 7:**
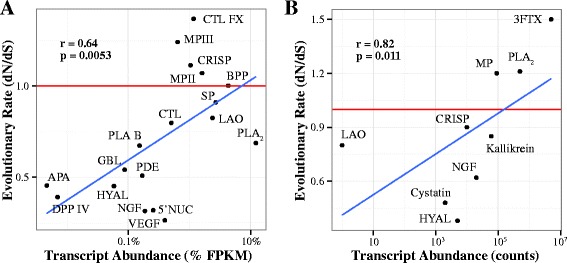
Data sets from two snake families show that more abundant venom proteins evolve more rapidly. Both panels show evolutionary rates, averaged by toxin class, as a function of abundance, using data from five crotaline species in three Old and New World genera (**a**), and the king cobra genome (re-analyzed from Fig. 5 in Vonk *et al.* [43]) (**b**). These results mirror the pairwise differences between *P. elegans* and *P. flavoviridis* shown in Fig. 6. In both cases, average evolutionary rates for the most abundant toxin classes tend to lie above dN/dS > 1, suggesting positive selection. Analyses for the two families are presented separately, because they vary greatly in toxin class composition and relative abundance of venom constituents. Significance of the associations was computed using Spearman’s rank correlation. Abbreviations: 3FTX: 3-finger toxins, APA: Aminopeptidase A, BPP: bradykinin-potentiating peptide, GBL: galactose-binding lectin, CRISP: cysteine-rich secretory proteins, DPP: dipeptidylpeptidase IV, HYAL: hyaluronidase, NGF: nerve growth factor, VEGF: vascular endothelial growth factor (see additional abbreviations in Fig. 6)

Patterns observed in the two *Protobothrops* species appear to hold generally true for crotaline snakes, based on data from three more species in two additional genera (Fig. 7a). Furthermore, the recently published genome of the king cobra (Family Elapidae) [43], shows the same pattern (Fig. 7b). These data from four additional studies, employing a range of methodologies, also show that more abundant snake venom proteins evolve faster. It is also possible that other organisms that depend on proteinaceous secretions for parasitism, competition, and defense may exhibit similar patterns, though further work will be necessary to test the generality of this conclusion, evident in snakes.

## Conclusions

The findings that venom protein concentrations can change dramatically over relatively short periods of time, as in the case of myotoxic PLA_2_s (Fig 3), and that relative concentration affects evolutionary rates of venom constituents, helps us to understand the trade-offs involved in venom formulation. Venoms are costly for snakes to produce [114]. Given that venom components can range in abundance by 3–5 orders of magnitude [42] (Fig. 5b), their cost to the snake likely also varies greatly, with more abundant components being more costly. Optimal foraging considerations suggest that snakes should invest in venom constituents in proportion to the benefit each of them provides. Therefore, variability in efficacy of the more abundant components will have the greatest fitness consequences for the snake, and those components should experience higher levels of selection. The higher evolutionary rates we observe in more abundant components are the evolutionary signature of this process. This also suggests that changes in venom component levels, rather than protein sequence-level changes, may be the most rapid and effective way for snakes to exploit new prey species.

## Deposition of data

Sequences of genes confirmed by mass spectrometry were curated manually by comparison with data publicly available at NCBI GenBank, and deposited in the DNA Data Bank of Japan under accession numbers AB984571-AB984614 for *P. elegans* and AB985221-AB985266 for *P. flavoviridis*.

## Methods

### Collection of snakes and extraction of venoms

Three *Protobothrops flavoviridis*, three *P. elegans*, and two putative hybrids were employed in this study. These specimens were captured in Nanjo City, Itoman City, and Yaese Town in southern Okinawa as part of a prefectural program to remove habus from densely inhabited areas. All animals, six males and two females, were adults, ranging from 82 to 150 cm total length (Table 1). Snakes were maintained at the Okinawa Prefectural Institute of Health and the Environment (OPIHE) after capture. Venoms were manually extracted into plastic beakers, transferred to cryovials, fast frozen on dry ice, and then stored at -30 °C until use.

### Removal of venom glands

Four days after venom extraction, one animal of each species was euthanized under pentobarbital anesthesia (>100 mg/kg), in accordance with OIST animal care protocols. Right and left venom glands and underlying skeletal muscle were excised, placed individually into 2 mL cryovials, flash frozen in liquid nitrogen, and stored at -80 °C until use.

### Transcriptomics

#### Isolation of total mRNA from venom glands and first strand cDNA synthesis

RNA was isolated using Qiagen RNeasy Plus Mini Kits. One μg of total RNA was used for first strand cDNA synthesis. ERCC ExFold RNA Spike-In Mix (Life Technologies) was included in the cDNA synthesis reaction according to the manufacturer’s protocol. To the RNA and spike-in mix, 2 μL of 10 μM poly T_START oligo 5′-AATTGCAGTGGTATCAACGCAGAGCGGCCGCTTTTTTTTTTTTTTTTTTTTTTTTTTTTTVN were added. The 9-μL mixture was incubated at 65 °C for 3 min, and then chilled on ice. The 11-μL reaction, containing 4 μL of 5× first strand synthesis buffer (Invitrogen), 1 μL of 10 mM dNTP (Promega), 2 μL of 0.1 M DTT (Invitrogen), 2 μL of 12 μM template-switching RNA oligo (5′-AAGCAGUGGUAUCAACGCAGAGUACAUGGG), 1 μL RNase inhibitor (Qiagen), and 1 μL Superscript II Reverse Transcriptase (Invitrogen) were added to each sample. Reactions were incubated at 42 °C for 60 min and the enzyme was heat inactivated at 65 °C for 15 min. 80 μL MilliQ water were added to each cDNA reaction.

#### Second strand cDNA synthesis

Second strand cDNA was synthesized with a limited number of PCR cycles. The 50-μL PCR reaction consisted of 1× Phusion HF buffer (Thermo Scientific), 200 μM dNTP (Promega), 0.5 μM START primer (5′-CGCCAGGGTTTTCCCAGTCACGACAATTGCAGTGGTATCAACGCAGA), 0.5 μM TS_long primer (5′-CTTGTAGGTTAAGTGGAGAGCTAACAATTTCACACAGGAAAGCAGTGGTATCAACGC), 0.5 μL of 2 U/μL Phusion DNA polymerase (Thermo Scientific), and 10 μL diluted cDNA. Twelve 50-μL PCR reactions were set up for each cDNA sample. PCR was carried out with the following conditions: initial denaturation at 98 °C for 30 s, with 8 cycles of denaturation at 98 °C for 10 s, 68 °C for 6 min, followed by final extension at 72 °C for 10 min. PCR reactions were concentrated with an Amicon Ultra-0.5 column (Millipore).

Concentrated PCR products were purified by solid phase reversible immobilization [119] using Dynabeads MyOne Carboxylic Acid (Invitrogen). 17 % PEG was used for purification. 1 ng of ds-cDNA was used library preparation and sequencing, using a Nextera XT DNA sample kit (illumina) according to manufacturer’s directions. Ten cycles of PCR were used for library amplification. Pooled libraries were sequenced paired-end for 250 cycles on an illumina Miseq system.

### Proteomics

#### Reduction and alkylation of snake venoms prior to enzymatic digestion

Crude venoms were gently vortexed for several seconds to suspend microsomes. Reduction was accomplished by adding 36 μL 8 M urea, 2 μL venom, 2 μL 500 mM DTT in ultrapure water, and 10 μL 500 mM of ammonium bicarbonate to 200 μL PCR tubes. Tubes were incubated at 60 °C for 45 min in an MJ PTC 200 Peltier thermocycler. Then, 4 μL 500 mM sodium iodoacetate were added to each tube and mixed gently. Tubes were incubated at 37 °C for 30 min in the dark. Afterward, an additional 2 μL 500 mM DTT were added to quench the alkylation reaction. Sample desalting was done by pipetting the reaction from the PCR tubes into 10K Nanosep centrifugal filters (Pall Corp.) and centrifuging them at 18,407 × g for 25 min. Samples were rehydrated with 50 μL of 50 mM ammonium bicarbonate and were again centrifuged to further de-salt the samples.

#### Tryptic hydrolyses

After the second centrifugation, 10 μL 8 M urea were added to the membrane and the membrane surface was wetted with the urea by rotating the tube manually. Then 90 μL 50 mM ammonium bicarbonate were added to the membrane and the filter was vortexed gently to re-suspend de-salted proteins. Solutions were pipetted from the filter and placed in clean 200 μL PCR tubes. Finally 10 μg of trypsin (Pierce 90055) dissolved in 10 μL 1 mM HCl were added to each tube. Tubes were incubated 24 h at 37 °C. Then, samples were pipetted back into their original 10K Nanosep filters and centrifuged again at 18,407 × g for 30 min to collect the de-proteinated filtrate which was frozen at -30 °C until analysis by mass spectrometry.

#### Chymotryptic hydrolyses

The procedure for chymotrypsin (Pierce 90056) was nearly identical to that for trypsin, except that after wetting the Nanosep filter with 10 μL 8 M urea, 10 μL 50 mM ammonium bicarbonate, 5 μL 200 mM CaCl_2_, and 65 μL ultrapure water were added to the membrane. All subsequent procedures were identical to those for trypsin.

#### Formic acid hydrolyses

Crude venoms were gently vortexed for few seconds. Digestion of proteins was done by mixing 2 μL venom, 4 μL 500 mM DTT in ultrapure water and 94 μL 2 % formic acid (Wako 063-04192) in 200 μL PCR tubes. Tubes were incubated for 2 h at 99 °C and then frozen at -30 °C until analysis by mass spectrometry.

#### Protein sequencing

For each sample, 3 technical repeats were run for each of the three hydrolyses; thus each sample was analyzed nine times. For each mass spectrogram, 1 μL was injected into a Waters nanoACQUITY UPLC Class-M with a Waters SYNAPT G2-S High Definition Mass Spectrometer equipped with Ion Mobility in tandem. Peptides were trapped in a nanoACQUITY 2G-V/MTrap 5 μm Symmetry C_18_ trap column at 5 μL/min, and separated in a nanoACQUITY UPLC 1.7 μm BEH130 C_18_ 75 μm × 200 mm analytical column at 300 nL/min, 35 °C, with a gradient of 3–60 % B in 90 min. The column was equilibrated in 0.1 % formic acid in water and the elution buffer (B) was 0.1 % formic acid in acetonitrile. Mass spectrometer settings were as follows: 1.8 kV spray voltage, cone voltage 30 V, acquisition mode fast-DDA with ion mobility separation, resolution 25,000 FWHM, continuum mode, mass range 100–2,000 m/z, acquisition rate 1 Hz/function, quad profile manual using first mass 400, 25 % dwell time, and a 25 % scan time ramp, second mass 600 with 25 % dwell time and third mass 800, collision energy ramped from 25 to 45 eV, and ion mobility wave velocity 1,200 m/s. The mass spectrometer was calibrated with Glu-fibrinopeptide B 100 fmol/uL in 50 % methanol in water. The same solution was used as a lock mass at 1 μL/min. The top eight most intense ions from each full MS scan were fragmented in a data-dependent manner with CID, using an exclusion of 60 s.

### Bioinformatics

Analysis of gene expression, quantitative proteomics, and evolutionary rate correlations were performed using the R statistical package. Scripts performing the analysis, as well as the accompanying SQL tables are provided on DataDryad http://dx.doi.org/10.5061/dryad.3948r.

#### Transcriptome assembly, quantitation and validation

8.0 and 8.5 million read pairs comprising 3.6 and 3.7 gigabases were sequenced from the *P. elegans* and *P. flavoviridis* libraries, respectively. Sequences were quality and adaptor trimmed using Trimmomatic, with the SLIDINGWINDOW:4:30 MINLEN:65 parameters and Nextera adaptor sequences [120]. Reads were then assembled using the Trinity suite (r20131110) [121], and then re-mapped to the assembly using the RSEM (v1.2.13) pipeline, with bowtie2 as the mapper [122, 123]. Ninety nine and ninety eight percent of the reads could be mapped to the reference in this manner for *P. elegans* and *P. flavoviridis*, respectively. Venom transcripts in Fig 3 were manually curated individually, and compared to published sequences.

Fragments per kilobase mapped (FPKM) output from RSEM were used to determine the coverage cutoff for proteomic analysis and for analysis of relative transcript expression levels. To determine the lowest FPKM cutoff for the transcriptomic analysis, we used the relationship between expected and observed values for ERCC spike-in controls. As the cutoff increased, so did the strength of the correlation, reaching a maximum of 0.96 at FPKM 32. Assembled sequences more abundant than this cutoff were used as reference sequences for downstream proteomic analysis.

Well after the completion of this study, a new assembler, VTBuilder [124], was published, specifically designed to remedy the deficiencies of Trinity, which typically produces large numbers of incomplete transcripts with highly diversified protein families. We validated our unfiltered assembly and compared it against the new method by evaluating the assembly of spike-in transcripts. We found that VTBuilder detected only 18 and 34 of the 92 spike-in controls, for *P. elegans* and *P. flavoviridis*, respectively, vs. 73 and 82, assembled by Trinity. Furthermore, all but a few of the spike-ins were represented by multiple sequences, as many as 22, whereas virtually all Trinity assemblies matched a unique spike-in control, or produced at most two matches. This evaluation suggests that the Trinity assembly performed well for the known sequences in our data, which spanned a wide range of concentrations. All but one of the venom transcripts from Fig 3 could be found in the VTBuilder assembly (Pe_comp1091_c0_seq1, a low-abundance aminopeptidase was missing). VTBuilder suggested that five pairs of low-abundance Trinity transcripts assemblies were incompletely assembled by the latter (three in *P. elegans* and two in *P. flavoviridis*). In most cases, but not always, VTBuilder transcripts had better matches to NCBI protein data. In any case, even assuming the VTBuilder analysis was correct in its reconstruction, no more than one of these pairs had a homolog in the other transcriptome, so differences in assembly should not bias our estimates of evolutionary rates (see below).

#### Evolutionary rate analysis

For purposes of evolutionary rate analysis, proteins that had more than one high quality matching peptide from the venom proteome were classified as venom components; everything else was considered a tissue component. Likely coding sequences for transcripts passing the FPKM filter were determined using FrameDP [125]. Putative homologs were then selected among the predicted protein sequences using reciprocal best BLAST. Predicted protein sequence pairs were aligned using MUSCLE [126] and codon alignments were created using PAL2NAL [127]. For each pair of codon aligned sequences, pairwise evolutionary rates (the ratio of synonymous to non-synonymous substitutions) were computed using a maximum likelihood model implemented in PAML (parameters: runmode = -2, cleandata = 0, model = 1, NSsites = [0]) [128]. The R script containing the evolutionary rate analysis can be found in the Additional file 9 and an SQL database with the raw data has been deposited in DataDryad (see Data Accessibility section). The combination of the R script and database is sufficient to reproduce the entire analysis, starting after PAML computations.

We tested the generality of relationships between toxin abundance and evolution by combining results from the present study with those of three recent investigations that reported relative toxin abundances [42, 129, 130]. For each protein class, sequences greater than 150 base pairs were codon-aligned using Geneious (v. 8.0.5), with each alignment inspected manually for quality. Signal peptides were detected by comparison with published and annotated homologs and were removed. A tree for each protein alignment was then computed using RAXML with the GTRCAT model [131]. The free-ratio model implemented in codeml was then used to estimate evolutionary rates along all branches of each tree. As we were interested in only the most recent evolutionary events, for each protein we considered only the branch leading up to each leaf in the tree. We then removed any extreme values from the data set (dN/dS > 3), which typically resulted from a near total lack of synonymous substitutions occurring along some branches, and averaged both the evolutionary rates and expression levels in each class. We also computed a correlation between relative transcript abundance within a toxin class and its evolutionary rate using values previously reported for the king cobra genome project [43].

##### Detecting positive selection in *Protobothrops*

In order to determine whether the increased evolutionary rates were due to relaxed or to positive selection, we added publicly available *Protobothrops* sequences for genes showing evidence of accelerated evolution (dn/ds = 1). We found additional data for C-type lectins, PLA_2_s, metalloproteases, and serine proteases. Sequence alignments were tested for the presence of recombination breakpoints and partitioned using SBP, and then tested for positive or diversifying selection using PARRIS and MEME algorithms implemented on the DataMonkey server [132–136].

#### Protein identification

Acquisition of mass spectrometric data employed a Waters MassLynx v4.0. Chromatographic data was processed using Waters Progenesis QI for Proteomics for mass correction (using acquired lock mass data), alignment, normalization, isotope grouping, peak picking, and ion drift separation, using default values. For peptide identification, files of fragmentation spectra (~330,000 spectra), generated only from monoisotopic precursors, were exported in mgf format for analysis using Mascot (version 2.4), and Proteome Discoverer (version 1.2, using Sequest). Search parameters for both algorithms were: a maximum of two missed cleavages, with precursor and fragment mass tolerance set to 20 ppm and 0.3 Da, respectively. Carboxyamidomethylation of cysteine was set as a fixed modification, while methionine oxidation, asparagine and glutamine deamidation, and N-terminal acetylation were set as variable modifications for tryptic and chymotryptic digests. Asparagine and glutamine deamidation and formylation of peptide N-termini were set as variable modifications for formic acid digests. Reagents used for sequencing (trypsin, R and K; chymotrypsin, F, L, W, and Y; formic acid, D and M) were specified in each case.

A database from our transcript data was constructed using TransDecoder [137], http://transdecoder.github.io, version r20131110), using a minimum length open reading frame of 5 amino acids or more than 10 nucleotides, as parameters. These relaxed settings were chosen to avoid the loss of small natural peptides present in the venoms. The common Repository of Adventitious Proteins – cRAP (http://www.thegpm.org/crap/) was merged with this constructed database for searching (*Protobothrops elegans* plus cRAP = 116,501 entries; *Protobothrops flavoviridis* plus cRAP = 430,984 entries; *Protobothrops* hybrids plus cRAP = 237,041). Protein and peptide identifications from Mascot and Sequest were combined, setting the false discovery rate to 0.1 % at the peptide level. Proteins with one hit were manually verified in all replicates (chromatographic peak), filtering out those that did not appear in at least 80 % of the runs.

## Availability of supporting data

The R script containing the evolutionary rate analysis and an SQL database with the raw data used in the bioinformatic analysis, which contains raw mass spectrometry data, transcript counts, and other types of data, can be found on DataDryad: doi:10.5061/dryad.3948r The combination of the R script and database is sufficient to reproduce the entire analysis, starting after PAML computations.

## Additional files

Additional file 1: **Supplementary Table 3.**

Additional file 2: Table S1. Transcript details for the *Protobothrops elegans* transcriptome. Complete transcripts are highlighted in blue; incomplete transcripts are in yellow. Relative transcript abundance is provided in the column “FPKM %.” These data provide the most quantitative estimates of venom composition; however, some of the incomplete transcripts pertaining to the same protein families (e.g., metalloproteases) may, in fact, be the same protein, if the transcripts were completely nonoverlapping. (PDF 132 kb)

Additional file 3: Table S2. Transcript details for the *Protobothrops flavoviridis* transcriptome. Details as for Additional file 2: Table S1. (PDF 130 kb)

Additional file 4: Figure S1. Phospholipases A_2_ in venoms of *P. elegans*, *P. flavoviridis*, and hybrids between the two. **A.** Myotoxic PLA_2_s. Catalytic PLA_2_s have a critical Asp residue in position 48 (See panels B-D). All of these enzymes manifest a basic residue in this position, preventing the binding of a critical Ca^2+^ ion involved in catalysis. However, while most New World crotaline myotoxins have Lys at this position, *P. elegans* and some other Asian crotalines have substituted Arg. Partial transcript comp552_c0_seq1 from *P. flavoviridis* is also a myotoxin, but it is a minor venom component (Additional file 3: Table S2). Signal peptides are shown to the left of the vertical black line. **B.** Acidic PLA_2_s. These enzymes are strongly lipolytic and myotoxic, though much less myotoxic than the non-catalytic myotoxins shown in panel D. **C.** Moderately basic PLA_2_s. *P. flavoviridis* PLA_2_ 1 is most similar to PL-Y, PL-X, and PLA-B. Like those in panel B, these enzymes are strongly lipolytic and moderately myotoxic. PLA-B induces edema [12]. **D.** Weakly neurotoxic PLA_2_s. The partial transcript, *P. flavoviridis* comp48_c0_seq1 is identical, as far as can be seen to PLA-N(O) [11]. (PDF 140 kb)

Additional file 5: Figure S3. Mean frequency per specimen with which individual amino acid residues in the noncatalytic, myotoxic *P. elegans* PLA_2_, comp43_c0_seq1, were sequenced in *P. elegans* and hybrid venoms. Not only were most of the same peptides sequenced in these venoms, but they were sequenced with the same relative frequencies. (PDF 234 kb)

Additional file 6: Figure S5. Hybrids express most proteins found in the two parental species. Every colored node corresponds to a transcript and is color-coded by toxin family. The toxins are connected to black nodes labeled with species identities, by edges whose thickness corresponds to the protein expression level in that species. Expression values are given as a percentage of the total, averaged across all samples, and range from less than 0.001 to 19 %. More detail on expression levels of individual toxins is presented in Fig 3. Virtually all toxins are expressed in the hybrids, except for a few venom components, which are poorly expressed in the parental species, and may have been missed in the hybrids due stochasticity involved in mass spectrometric detection. (PNG 1018 kb)

Additional file 7: Figure S2. Manual identification of CID fragment ions from a composite spectrum using >40 MS2 spectra from the same chromatographic peak associated with *Protobothrops elegans* transcript comp103_c0_seq1. Annotation was performed using the PEAKS studio 7.0 de novo sequencing module, followed by a search with the same database used for Mascot searches, and manual annotation to ascertain correctness. This process confirmed the sequence of the peptide and that Frame 1 is correct frame for translation. (PDF 320 kb)

Additional file 8: Figure S4. Venom peptides sequenced from both parental taxa and hybrids pertaining to transcripts for the homologous, basic, catalytic PLA_2_s, *P. elegans* comp47_c0_seq1 and *P. flavoviridis* comp41_c0_seq1. Hybrids inherited both homologs. (PDF 12 kb)

Additional file 9: **Supplementary Material.** (DOCX 70 kb)

## References

[1] Fraser HB, Hirsh AE, Steinmetz LM, Scharfe C, Feldman MW (2002). Evolutionary rate in the protein interaction network. Science.

[2] Subramanian S, Kumar S (2004). Gene expression intensity shapes evolutionary rates of the proteins encoded by the vertebrate genome. Genetics.

[3] Drummond DA, Bloom JD, Adami C, Wilke CO, Arnold FH (2005). Why highly expressed proteins evolve slowly. Proc Natl Acad Sci U S A.

[4] Zhang J, He X (2005). Significant impact of protein dispensability on the instantaneous rate of protein evolution. Mol Biol Evol.

[5] Makino T, Gojobori T (2006). The evolutionary rate of a protein is influenced by features of the interacting partners. Mol Biol Evol.

[6] Liao BY, Zhang J (2008). Null mutations in human and mouse orthologs frequently result in different phenotypes. Proc Natl Acad Sci U S A.

[7] Luz H, Vingron M (2006). Family specific rates of protein evolution. Bioinformatics.

[8] Liao BY, Weng MP, Zhang J (2010). Impact of extracellularity on the evolutionary rate of mammalian proteins. Genome Biol Evol.

[9] Julenius K, Pedersen AG (2006). Protein evolution is faster outside the cell. Mol Biol Evol.

[10] Dean MD, Good JM, Nachman MW (2008). Adaptive evolution of proteins secreted during sperm maturation: an analysis of the mouse epididymal transcriptome. Mol Biol Evol.

[11] Winter EE, Goodstadt L, Ponting CP (2004). Elevated rates of protein secretion, evolution, and disease among tissue-specific genes. Genome Res.

[12] Van Valen L (1974). Molecular evolution as predicted by natural selection. J Mol Evol.

[13] Dawkins R, Krebs JR (1979). Arms races between and within species. Proc R Soc Lond B Biol Sci.

[14] Aird SD (2002). Ophidian envenomation strategies and the role of purines. Toxicon.

[15] Barchan D, Ovadia M, Kochva E, Fuchs S (1995). The binding site of the nicotinic acetylcholine receptor in animal species resistant to alpha-bungarotoxin. Biochemistry.

[16] Takacs Z, Wilhelmsen KC, Sorota S (2004). Cobra (Naja spp.) nicotinic acetylcholine receptor exhibits resistance to Erabu sea snake (Laticauda semifasciata) short-chain alpha-neurotoxin. J Mol Evol.

[17] Jansa SA, Voss RS (2011). Adaptive evolution of the venom-targeted vWF protein in opossums that eat pitvipers. PLoS ONE.

[18] Mackessy SP (1988). Venom ontogeny in the Pacific rattlesnakes *Crotalus viridis helleri* and *C. v. oreganus*. Copeia.

[19] Mackessy SP (1996). Characterization of the major metalloprotease isolated from the venom of the northern pacific rattlesnake, *Crotalus viridis oreganus*. Toxicon.

[20] McCue MD (2007). Prey envenomation does not improve digestive performance in western diamondback rattlesnakes (Crotalus atrox). J Exp Zool A Ecol Genet Physiol.

[21] Daltry JC, Wuster W, Thorpe RS (1996). Diet and snake venom evolution. Nature.

[22] Chijiwa T, Deshimaru M, Nobuhisa I, Nakai M, Ogawa T, Oda N (2000). Regional evolution of venom-gland phospholipase A2 isoenzymes of Trimeresurus flavoviridis snakes in the southwestern islands of Japan. Biochem J.

[23] Silva Junior NJ, Aird SD (2001). Prey specificity, comparative lethality and compositional differences of coral snake venoms. Comp Biochem Physiol C Toxicol Pharmacol.

[24] Gibbs HL, Mackessy SP (2009). Functional basis of a molecular adaptation: prey-specific toxic effects of venom from Sistrurus rattlesnakes. Toxicon.

[25] Li M, Fry BG, Kini RM (2005). Eggs-only diet: its implications for the toxin profile changes and ecology of the marbled sea snake (Aipysurus eydouxii). J Mol Evol.

[26] Li M, Fry BG, Kini RM (2005). Putting the brakes on snake venom evolution: the unique molecular evolutionary patterns of Aipysurus eydouxii (Marbled sea snake) phospholipase A2 toxins. Mol Biol Evol.

[27] Ogawa T, Oda N, Nakashima K, Sasaki H, Hattori M, Sakaki Y (1992). Unusually high conservation of untranslated sequences in cDNAs for Trimeresurus flavoviridis phospholipase A2 isozymes. Proc Natl Acad Sci U S A.

[28] Nakashima K, Nobuhisa I, Deshimaru M, Nakai M, Ogawa T, Shimohigashi Y (1995). Accelerated evolution in the protein-coding regions is universal in crotalinae snake venom gland phospholipase A2 isozyme genes. Proc Natl Acad Sci U S A.

[29] Nobuhisa I, Nakashima K, Deshimaru M, Ogawa T, Shimohigashi Y, Fukumaki Y (1996). Accelerated evolution of Trimeresurus okinavensis venom gland phospholipase A2 isozyme-encoding genes. Gene.

[30] Ogawa T, Nakashima K, Nobuhisa I, Deshimaru M, Shimohigashi Y, Fukumaki Y (1996). Accelerated evolution of snake venom phospholipase A2 isozymes for acquisition of diverse physiological functions. Toxicon.

[31] Chuman Y, Nobuhisa I, Ogawa T, Deshimaru M, Chijiwa T, Tan NH (2000). Regional and accelerated molecular evolution in group I snake venom gland phospholipase A2 isozymes. Toxicon.

[32] Alape-Giron A, Persson B, Cederlund E, Flores-Diaz M, Gutierrez JM, Thelestam M (1999). Elapid venom toxins: multiple recruitments of ancient scaffolds. Eur J Biochem.

[33] Fry BG, Wuster W, Kini RM, Brusic V, Khan A, Venkataraman D (2003). Molecular evolution and phylogeny of elapid snake venom three-finger toxins. J Mol Evol.

[34] Kini RM, Kini RM (1997). Phospholipase A2 – a complex multifunctional protein puzzle. Venom phospholipase A2 enzymes: structure, function and mechanism.

[35] Lynch VJ (2007). Inventing an arsenal: adaptive evolution and neofunctionalization of snake venom phospholipase A2 genes. BMC Evol Biol.

[36] Bohlen CJ, Chesler AT, Sharif-Naeini R, Medzihradszky KF, Zhou S, King D (2011). A heteromeric Texas coral snake toxin targets acid-sensing ion channels to produce pain. Nature.

[37] Deshimaru M, Ogawa T, Nakashima K, Nobuhisa I, Chijiwa T, Shimohigashi Y (1996). Accelerated evolution of crotalinae snake venom gland serine proteases. FEBS Lett.

[38] Ogawa T, Chijiwa T, Oda-Ueda N, Ohno M (2005). Molecular diversity and accelerated evolution of C-type lectin-like proteins from snake venom. Toxicon.

[39] Juarez P, Comas I, Gonzalez-Candelas F, Calvete JJ (2008). Evolution of snake venom disintegrins by positive Darwinian selection. Mol Biol Evol.

[40] Casewell NR, Wagstaff SC, Harrison RA, Renjifo C, Wuster W (2011). Domain loss facilitates accelerated evolution and neofunctionalization of duplicate snake venom metalloproteinase toxin genes. Mol Biol Evol.

[41] Sunagar K, Johnson WE, O’Brien SJ, Vasconcelos V, Antunes A (2012). Evolution of CRISPs associated with toxicoferan-reptilian venom and mammalian reproduction. Mol Biol Evol.

[42] Aird SD, Watanabe Y, Villar-Briones A, Roy MC, Terada K, Mikheyev AS (2013). Quantitative high-throughput profiling of snake venom gland transcriptomes and proteomes (Ovophis okinavensis and Protobothrops flavoviridis). BMC Genomics.

[43] Vonk FJ, Casewell NR, Henkel CV, Heimberg AM, Jansen HJ, McCleary RJ (2013). The king cobra genome reveals dynamic gene evolution and adaptation in the snake venom system. Proc Natl Acad Sci U S A.

[44] Kini RM, Chan YM (1999). Accelerated evolution and molecular surface of venom phospholipase A2 enzymes. J Mol Evol.

[45] Mebs D (2001). Toxicity in animals. Trends in evolution?. Toxicon.

[46] Kini RM, Chinnasamy A (2010). Nucleotide sequence determines the accelerated rate of point mutations. Toxicon.

[47] Angulo Y, Escolano J, Lomonte B, Gutierrez JM, Sanz L, Calvete JJ (2008). Snake venomics of Central American pitvipers: clues for rationalizing the distinct envenomation profiles of Atropoides nummifer and Atropoides picadoi. J Proteome Res.

[48] Jiménez-Porras JM (1964). Venom proteins of the Fer-de-lance, *Bothrops atrox*, from Costa Rica. Toxicon.

[49] Ohno M, Menez R, Ogawa T, Danse JM, Shimohigashi Y, Fromen C (1998). Molecular evolution of snake toxins: is the functional diversity of snake toxins associated with a mechanism of accelerated evolution?. Prog Nucleic Acid Res Mol Biol.

[50] Dias GS, Kitano ES, Pagotto AH, Sant’anna SS, Rocha MM, Zelanis A (2013). Individual variability in the venom proteome of juvenile Bothrops jararaca specimens. J Proteome Res.

[51] Guo P, Malhotra A, Li PP, Pook CE, Creer S (2007). New evidence on the phylogenetic position of the poorly known Asian pitviper Protobothrops kaulbacki (Serpentes : Viperidae : Crotalinae) with a redescription of the species and a revision of the genus Protobothrops. Herpetol J.

[52] Katsuren S, Nishimura M, Nagata E, Ohama M. Numbers of Snakes Arriving Alive in Mass into Okinawa by Traders. Report by Okinawa Prefectural Institute of Health and Environment 1996, 30:133-136.

[53] Nishimura M, Akamine H. Dispersal Range of an Alien Viperid Snake, *Trimeresurus elegans* Established in Southern Okinawa Island after the Escape in 1976 - Results of Preliminary Studies in 2002. Annual Report of Okinawa Prefectural Institute of Health and Environment 2002, 36:89-92.

[54] Terada K (2011). The distribution, population density and controls of Protobothrops mucrosquamatus, Protobothrops elegans, Elaphe taeniura friesei, three snake species established on Okinawa Island. Bull Herpetol Soc Jpn.

[55] Ota H (1998). Geographic patterns of endemism and speciation in amphibians and reptiles of the Ryukyu archipelago, Japan, with special reference to their paleogeographical implications. Res Popul Ecol.

[56] Ota H (2000). The current geographic faunal pattern of reptiles and amphibians of the Ryukyu archipelago and adjacent regions. Tropics.

[57] Osozawa S, Su Z-H, Oba Y, Yagi T, Watanabe Y, Wakabayashi J (2013). Vicariant speciation due to 1.55 Ma isolation of the Ryukyu islands, Japan, based on geological and GenBank data. Entomol Sci.

[58] Wuster W, Peppin L, Pook CE, Walker DE (2008). A nesting of vipers: Phylogeny and historical biogeography of the Viperidae (Squamata: Serpentes). Mol Phylogenet Evol.

[59] Zhao K, Zhou Y, Lin Z (2000). Structure of basic phospholipase A2 from Agkistrodon halys Pallas: implications for its association, hemolytic and anticoagulant activities. Toxicon.

[60] Fraenkel-Conrat H (1982). Snake venom neurotoxins related to phospholipase A2. J Toxicol-Toxin Rev.

[61] Aird SD, Kaiser II (1985). Comparative studies on three rattlesnake toxins. Toxicon.

[62] Boldrini-França J, Rodrigues RS, Fonseca FP, Menaldo DL, Ferreira FB, Henrique-Silva F (2009). *Crotalus durissus collilineatus* venom gland transcriptome: analysis of gene expression profile. Biochimie.

[63] Tsai IH, Lu PJ, Wang YM, Ho CL, Liaw LL (1995). Molecular cloning and characterization of a neurotoxic phospholipase A2 from the venom of Taiwan habu (Trimeresurus mucrosquamatus). Biochem J.

[64] Kini RM, Evans HJ (1989). A model to explain the pharmacological effects of snake venom phospholipases A2. Toxicon.

[65] Kini RM (2003). Excitement ahead: structure, function and mechanism of snake venom phospholipase A2 enzymes. Toxicon.

[66] Cameron DL, Tu AT (1977). Characterization of myotoxin a from the venom of prairie rattlesnake (Crotalus viridis viridis). Biochemistry.

[67] Francis B, Gutierrez JM, Lomonte B, Kaiser II (1991). Myotoxin II from Bothrops asper (Terciopelo) venom is a lysine-49 phospholipase A2. Arch Biochem Biophys.

[68] Cintra AC, Marangoni S, Oliveira B, Giglio JR (1993). Bothropstoxin-I: amino acid sequence and function. J Protein Chem.

[69] Angulo Y, Chaves E, Alape A, Rucavado A, Gutierrez JM, Lomonte B (1997). Isolation and characterization of a myotoxic phospholipase A2 from the venom of the arboreal snake Bothriechis (Bothrops) schlegelii from Costa Rica. Arch Biochem Biophys.

[70] Ikeda N, Chijiwa T, Matsubara K, Oda-Ueda N, Hattori S, Matsuda Y, et al. Unique structural characteristics and evolution of a cluster of venom phospholipase A(2) isozyme genes of Protobothrops flavoviridis snake. Gene. 2010. 10.1016/j.gene.2010.04.001 20406671

[71] Kinjoh K, Kosugi T, Nakamura M, Hanashiro K, Sunagawa M, Tokeshi Y (1997). Habutobin splits the Arg16-Gly17 bond in the A alpha chain of rabbit fibrinogen. Thromb Haemost.

[72] Nejime T, Kinjoh K, Nakamura M, Hanashiro K, Sunagawa M, Eguchi Y (2000). Habutobin recognizes Thr(7) in the sequence of fibrinopeptide A of rabbit fibrinogen. Toxicon.

[73] Nakamura M, Kinjoh K, Miyagi C, Oka U, Sunagawa M, Yamashita S (1995). Pharmacokinetics of habutobin in rabbits. Toxicon.

[74] Sunagawa M, Hanashiro K, Nakamura M, Kosugi T (1996). Habutobin releases plasminogen activator (U-PA) from bovine pulmonary artery endothelial cells. Toxicon.

[75] Yamamoto C, Tsuru D, Oda-Ueda N, Ohno M, Hattori S, Kim ST (2002). Flavoxobin, a serine protease from Trimeresurus flavoviridis (habu snake) venom, independently cleaves Arg726-Ser727 of human C3 and acts as a novel, heterologous C3 convertase. Immunology.

[76] Zhang Y, Wisner A, Xiong Y, Bon C (1995). A novel plasminogen activator from snake venom. Purification, characterization, and molecular cloning. J Biol Chem.

[77] Gladner JA, Murtaugh PA, Folk JE, Laki K (1963). Nature of peptides released by thrombin. Ann N Y Acad Sci.

[78] Osbahr AJ, Colman RW, Gladner JA, Laki K (1964). The nature of the peptides released from canine fibrinogen. Biochem Biophys Res Commun.

[79] Rojnuckarin P, Intragumtornchai T, Sattapiboon R, Muanpasitporn C, Pakmanee N, Khow O (1999). The effects of green pit viper (Trimeresurus albolabris and Trimeresurus macrops) venom on the fibrinolytic system in human. Toxicon.

[80] Markland FS, Kettner C, Schiffman S, Shaw E, Bajwa SS, Reddy KN (1982). Kallikrein-like activity of crotalase, a snake venom enzyme that clots fibrinogen. Proc Natl Acad Sci U S A.

[81] Zaganelli GL, Zaganelli MG, Magalhaes A, Diniz CR, de Lima ME (1996). Purification and characterization of a fibrinogen-clotting enzyme from the venom of jararacucu (Bothrops jararacussu). Toxicon.

[82] Serrano SM, Hagiwara Y, Murayama N, Higuchi S, Mentele R, Sampaio CA (1998). Purification and characterization of a kinin-releasing and fibrinogen-clotting serine proteinase (KN-BJ) from the venom of Bothrops jararaca, and molecular cloning and sequence analysis of its cDNA. Eur J Biochem.

[83] Komori Y, Tatematsu R, Tanida S, Nikai T (2001). Thrombin-like enzyme, flavovilase, with kinin-releasing activity from Trimeresurus flavoviridis (habu) venom. J Nat Toxins.

[84] Jia YH, Jin Y, Lu QM, Li DS, Wang WY, Xiong YL (2003). Jerdonase, a novel serine protease with kinin-releasing and fibrinogenolytic activity from Trimeresurus jerdonii venom. Sheng Wu Hua Xue Yu Sheng Wu Wu Li Xue Bao (Shanghai).

[85] Oyama E, Takahashi H (2006). Substrate specificity of two thrombin like enzymes (elegaxobin, elegaxobin II) from the venom of Trimeresurus elegans (Sakishima-habu), using neutralizing antibody. Toxicon.

[86] Bajwa SS, Markland FS, Russell FE (1980). Fibrinolytic enzyme(s) in western diamondback rattlesnake (Crotalus atrox) venom. Toxicon.

[87] Retzios AD, Markland FS (1988). A direct-acting fibrinolytic enzyme from the venom of Agkistrodon contortrix contortrix: effects on various components of the human blood coagulation and fibrinolysis systems. Thromb Res.

[88] Wei WL, Sun JJ, Chen JS (1996). Synergism of procoagulation effect of thrombin-like enzymes from Deinagkistrodon acutus and Agkistrodon halys snake venoms. Zhongguo Yao Li Xue Bao.

[89] Santoro ML, Sano-Martins IS (1993). Different clotting mechanisms of Bothrops jararaca snake venom on human and rabbit plasmas. Toxicon.

[90] Pirkle H, Markland FS, Theodor I (1976). Thrombin-like enzymes of snake venoms: actions on prothrombin. Thromb Res.

[91] Swenson S, Markland FS (2005). Snake venom fibrin(ogen)olytic enzymes. Toxicon.

[92] Kamiguti AS, Cardoso JL (1989). Haemostatic changes caused by the venoms of South American snakes. Toxicon.

[93] Hoover-Plow J (2010). Does plasmin have anticoagulant activity?. Vasc Health Risk Manag.

[94] Fox JW, Serrano SM (2005). Structural considerations of the snake venom metalloproteinases, key members of the M12 reprolysin family of metalloproteinases. Toxicon.

[95] Maeno H (1962). Biochemical analysis of pathological lesions caused by habu snake venom with special reference to hemorrhage. J Biochem.

[96] Yoshikura H, Ogawa H, Osaka A, Omori-Sato T (1966). Action of Trimeresurus flavoviridis venom and the partially purified hemorrhagic principles on animal cells cultivated *in vitro*. Toxicon.

[97] Homma M, Kosuge T, Okonogi T, Hattori Z, Sawai Y (1967). A histopathological study on arterial lesions caused by Habu (Trimeresurus flavoviridis) venom. Jpn J Exp Med.

[98] Tsuchiya M, Oshio C, Ohashi M, Fujishiro Y (1975). Cinematographic analysis of the hemorrhage induced by the venom of Trimeresurus flavoviridis. Bibl Anat.

[99] Omori-Satoh T, Ohsaka A (1970). Purification and some properties of hemorrhagic principle I in the venom of Trimeresurus flavoviridis. Biochim Biophys Acta.

[100] Takahashi T, Osaka A (1970). Purification and some properties of two hemorrhagic principles (HR2a and HR2b) in the venom of Trimeresurus flavoviridis; complete separation of the principles from proteolytic activity. Biochim Biophys Acta.

[101] Omori-Satoh T, Sadahiro S (1978). Resolution of the main hemorrhagic principle of Trimeresurus flavoviridis venom into two parts, HRIA and HRIB [proceedings]. Jpn J Med Sci Biol.

[102] Nikai T, Niikawa M, Komori Y, Sekoguchi S, Sugihara H (1987). Proof of proteolytic activity of hemorrhagic toxins, HR-2a and HR-2b, from Trimeresurus flavoviridis venom. Int J Biochem.

[103] Omori-Satoh T, Sadahiro S (1979). Resolution of the major hemorrhagic component of Trimeresurus flavoviridis venom into two parts. Biochim Biophys Acta.

[104] Takeya H, Oda K, Miyata T, Omori-Satoh T, Iwanaga S (1990). The complete amino acid sequence of the high molecular mass hemorrhagic protein HR1B isolated from the venom of Trimeresurus flavoviridis. J Biol Chem.

[105] Yonaha K, Iha M, Tomihara Y, Nozaki M, Yamakawa M (1991). Characterization of three hemorrhagic factors from the venom of Okinawa habu (Trimeresurus flavoviridis). Toxicon.

[106] Kishimoto M, Takahashi T (2002). Molecular cloning of HR1a and HR1b, high molecular hemorrhagic factors, from Trimeresurus flavoviridis venom. Toxicon.

[107] Masuda S, Hayashi H, Atoda H, Morita T, Araki S (2001). Purification, cDNA cloning and characterization of the vascular apoptosis-inducing protein, HV1, from Trimeresurus flavoviridis. Eur J Biochem.

[108] Masuda S, Ohta T, Kaji K, Fox JW, Hayashi H, Araki S (2000). cDNA cloning and characterization of vascular apoptosis-inducing protein 1. Biochem Biophys Res Commun.

[109] Wu WB, Chang SC, Liau MY, Huang TF (2001). Purification, molecular cloning and mechanism of action of graminelysin I, a snake-venom-derived metalloproteinase that induces apoptosis of human endothelial cells. Biochem J.

[110] Atoda H, Morita T (1989). A novel blood coagulation factor IX/factor X-binding protein with anticoagulant activity from the venom of Trimeresurus flavoviridis (Habu snake): isolation and characterization. J Biochem.

[111] Matsuzaki R, Yoshiara E, Yamada M, Shima K, Atoda H, Morita T (1996). cDNA cloning of IX/X-BP, a heterogeneous two-chain anticoagulant protein from snake venom. Biochem Biophys Res Commun.

[112] Fisher RA (1930). The genetical theory of natural selection.

[113] Bieber AL, Lee C-Y (1979). Metal and nonprotein constituents in snake venoms. Snake venoms, vol. 52.

[114] McCue MD, Mason R (2006). Cost of producing venom in three North American pitviper species. Copeia.

[115] Nakashima K, Ogawa T, Oda N, Hattori M, Sakaki Y, Kihara H (1993). Accelerated evolution of Trimeresurus flavoviridis venom gland phospholipase A2 isozymes. Proc Natl Acad Sci U S A.

[116] Krylov DM, Wolf YI, Rogozin IB, Koonin EV (2003). Gene loss, protein sequence divergence, gene dispensability, expression level, and interactivity are correlated in eukaryotic evolution. Genome Res.

[117] Hahn MW, Kern AD (2005). Comparative genomics of centrality and essentiality in three eukaryotic protein-interaction networks. Mol Biol Evol.

[118] Sasa M (1999). Diet and snake venom evolution: can local selection alone explain intraspecific venom variation?. Toxicon.

[119] Tin MM, Economo EP, Mikheyev AS (2014). Sequencing degraded DNA from non-destructively sampled museum specimens for RAD-tagging and low-coverage shotgun phylogenetics. PLoS ONE.

[120] Bolger AM, Lohse M, Usadel B (2014). Trimmomatic: a flexible trimmer for Illumina sequence data. Bioinformatics.

[121] Grabherr MG, Haas BJ, Yassour M, Levin JZ, Thompson DA, Amit I (2011). Full-length transcriptome assembly from RNA-Seq data without a reference genome. Nat Biotechnol..

[122] Li H (2011). A statistical framework for SNP calling, mutation discovery, association mapping and population genetical parameter estimation from sequencing data. Bioinformatics.

[123] Langmead B, Salzberg SL (2012). Fast gapped-read alignment with Bowtie 2. Nat Methods.

[124] Archer JP, Whiteley G, Casewell NR, Harrison RA, Wagstaff SC (2014). VTBuilder: a tool for the assembly of multi isoform transcriptomes. BMC Bioinformatics.

[125] Gouzy J, Carrere S, Schiex T (2009). FrameDP: sensitive peptide detection on noisy matured sequences. Bioinformatics.

[126] Edgar RC (2004). MUSCLE: multiple sequence alignment with high accuracy and high throughput. Nucleic Acids Res.

[127] Suyama M, Torrents D, Bork P (2006). PAL2NAL: robust conversion of protein sequence alignments into the corresponding codon alignments. Nucleic Acids Res.

[128] Yang Z (2007). PAML 4: phylogenetic analysis by maximum likelihood. Mol Biol Evol.

[129] Rokyta DR, Lemmon AR, Margres MJ, Aronow K (2012). The venom-gland transcriptome of the eastern diamondback rattlesnake (Crotalus adamanteus). BMC Genomics.

[130] Rokyta DR, Wray KP, McGivern JJ, Margres MJ (2015). The transcriptomic and proteomic basis for the evolution of a novel venom phenotype within the Timber Rattlesnake (Crotalus horridus). Toxicon.

[131] Stamatakis A (2006). RAxML-VI-HPC: maximum likelihood-based phylogenetic analyses with thousands of taxa and mixed models. Bioinformatics.

[132] Pond SL, Frost SD (2005). Datamonkey: rapid detection of selective pressure on individual sites of codon alignments. Bioinformatics.

[133] Kosakovsky Pond SL, Posada D, Gravenor MB, Woelk CH, Frost SD (2006). Automated phylogenetic detection of recombination using a genetic algorithm. Mol Biol Evol.

[134] Scheffler K, Martin DP, Seoighe C (2006). Robust inference of positive selection from recombining coding sequences. Bioinformatics.

[135] Delport W, Poon AF, Frost SD, Kosakovsky Pond SL (2010). Datamonkey 2010: a suite of phylogenetic analysis tools for evolutionary biology. Bioinformatics.

[136] Murrell B, Wertheim JO, Moola S, Weighill T, Scheffler K, Kosakovsky Pond SL (2012). Detecting individual sites subject to episodic diversifying selection. PLoS Genet.

[137] Haas BJ, Papanicolaou A, Yassour M, Grabherr M, Blood PD, Bowden J (2013). De novo transcript sequence reconstruction from RNA-seq using the Trinity platform for reference generation and analysis. Nat Protoc.

