# Supplementary material for: Snake venoms are integrated systems, but abundant venom proteins evolve more rapidly
Source: BMC Genomics. 2015 Aug 28;16:647. doi: 10.1186/s12864-015-1832-6 (PMC4552096; doi:10.1186/s12864-015-1832-6)
Supplement: Supplementary file 2 — Transcript details for the Protobothrops elegans transcriptome. Complete transcripts are highlighted in blue; incomplete transcripts are in yellow. Relative transcript abundance is provided in the column “FPKM %.” These data provide the most quantitative estimates of venom composition; however, some of the incomplete transcripts pertaining to the same protein families (e.g., metalloproteases) may, in fact, be the same protein, if the transcripts were completely nonoverlapping. (PDF 132 kb) [file 12864_2015_1832_MOESM2_ESM.pdf]

ID	DDBJ Accession #	Transcript Toxin Class	FPKM	% FPKM	Transcript Length (bp)	Transcript Length (AA encoded)	5'-UTR (AA)	Signal Peptide (AA)	Prepro-peptide	Transcribed Length (Total AA - 5'UTR)	Translated Length (Total AA - (UTR+SP))	# of Unique Peptides Sequenced	# of Amino Acids Sequenced by MS	Peptide Coverage AA %	Nearest BLAST Hit	Species of Nearest BLAST Hit	Open Reading Frame	Frame
comp43_c0_seq1	AB984571	Phospholipase A_2 1	606,214	73.4%	792	264	1-12	13-28	None	137	121	35	115	95.0%	AB219807.1	*Protobothrops elegans*	*RGLSIPRSGFGRMRTLWIMAVLLLGVEGSLIELWKMVF	Frame 1 Reverse
comp44_c1_seq1	AB984572	Serine Protease 1	45,388	5.5%	4,744	1,581	1-16	17-34	None	257	239	39	182	76.2%	X83224.1	*Protobothrops mucrosquamatus*	*GTGILQANSLLRSVEAMVLIRVLANLLILQLSYAQKSSE	Frame 2
comp46_c0_seq1	AB984573	Metalloprotease P-II 1	39,907	4.8%	2,029	676	1-37	38-57	None	412	392	62	216	55.1%	HQ731070.1	*Protobothrops mucrosquamatus*	*MKLKDSFLASHRQQRKSSGWLESRKRLPVFQPNPASK	Frame 2
comp47_c0_seq1	AB984574	Phospholipase A_2 2	29,290	3.5%	434	144	Missing	1-8	None	130	122	31	85	69.7%	Q2PG83.1	*Protobothrops elegans*	VLLLGVEGGLWQFENMIMKVAKKSGILSYSAYGCYCG	Frame 1
comp54_c0_seq1	AB984575	BPP-CNP	11,814	1.4%	919	306	Missing	Missing	None	34	34	1	10	29.4%	AB749765.1	*Protobothrops elegans*	GGARRLKGLGKKAVGEGCFGLKLDRIGSTSGLGC*	Frame 2
comp48_c0_seq1	AB984576	Serine Protease 2	11,513	1.4%	250	83	Missing	Missing	None	83	83	6	40	48.2%	AB848166.1	*Protobothrops flavoviridis*	INEHRSLVVLFNSSGALCGGTLINQEWVLTAAHCDMPN	Frame 1
comp63_c0_seq1	AB984577	Serine Protease 3	9,872	1.2%	214	71	Missing	Missing	None	71	71	10	50	70.4%	AB848157.1	*Protobothrops flavoviridis*	WVLTAAHCDRSSIYMYIGMHDKKVTFDDEQGRSPKEK	Frame 1
comp52_c0_seq1	AB984578	Serine Protease 4	9,371	1.1%	221	73	Missing	Missing	None	73	73	15	53	72.6%	AAD01624.1	*Gloydius brevicaudus*	EHRSLVVFFNSSGVFCGGTLINKEWVLTAARCDSKNFQ	Frame 2
comp49_c0_seq1	AB984579	Serine Protease 5	6,814	0.8%	206	68	Missing	Missing	None	54	54	7	48	88.9%	AB848164.1	*Protobothrops flavoviridis*	GGPLICNGEIQGIVSWGGDICAQPHEPGHYTKVFYYIDW	Frame 1
comp53_c0_seq1	AB984580	Metalloprotease P-IIa 2	6,726	0.8%	302	100	Missing	Missing	Missing	83	83	10	48	57.8%	AB059572.1	*Protobothrops elegans*	HQHTACCCRPFQLHCWLGMEGISGTVTRYAIAVIIQVIT	Frame 3
comp60_c0_seq1	AB984581	Metalloprotease P-IIIa 1	6,248	0.8%	625	208	Missing	Missing	Missing	200	200	21	138	69.0%	AB665726.1	*Protobothrops flavoviridis*	EDCDCGSPATCRDRCCNAATCKLRQGAQCAEGLCCYQ	Frame 1 Reverse
comp57_c0_seq1	AB984582	Metalloprotease P-IIIc 2	5,924	0.7%	539	179	Missing	Missing	Missing	179	179	21	145	81.0%	AB051849.1	*Viridovipera stejnegeri*	QCAEGLCCDQCRFKAAGTECRAATDECDMADLCTGRS	Frame 1
comp70_c0_seq1	AB984583	VEGF	4,405	0.5%	1,261	420	1-1	2-25	None	143	119	14	92	77.3%	Q330K6.1	*Protobothrops mucrosquamatus*	*AMAAYLLAVAILFCIQGWPSGTVQGQVMPFMEVYDRS	Frame 1
comp68_c0_seq1	AB984584	LAO	4,295	0.5%	2,862	954	1-22	23-39	None	505	488	71	397	81.4%	AB848142.1	*Protobothrops flavoviridis*	*ALCLASVTFLPSIAIHSLQANKMNVFFMFSLLFLAALG	Frame 3
comp67_c0_seq1	AB984585	Serine Protease 6	3,708	0.4%	227	75	Missing	Missing	None	75	75	2	38	50.7%	AB851961.1	*Protobothrops flavoviridis*	INEHRSLALVYITSGFLCGGTLIHPEWVMTAAHCDRGNI	Frame 3
comp69_c0_seq1	AB984586	Metalloprotease P-IIIb 3	3,517	0.4%	538	179	Missing	Missing	Missing	159	159	6	59	37.1%	AB074144.1	*Protobothrops flavoviridis*	AGTECRAAESECDIPENCTGQSAECPTDRFHKNGLPCLY	Frame 1
comp75_c0_seq1	AB984587	Metalloprotease P-IIIc 4	2,976	0.4%	519	173	Missing	Missing	Missing	173	173	23	116	67.1%	AB051849.1	*Protobothrops flavoviridis*	WESDEPIKEDSQSNLTPAQQKYLDAKKYVKFFLVADHI	Frame 1 Reverse
comp77_c0_seq1	AB984588	Metalloprotease P-IIIc 5	2,262	0.3%	226	75	Missing	Missing	Missing	74	74	6	30	40.5%	DQ335449.1	*Viridovipera stejnegeri*	KDTCTCRAKACVMAGTLSCEASLLFSDCSRQEHRAFLI	Frame 3 Reverse
comp76_c0_seq1	AB984589	Metalloprotease P-III 6	2,108	0.3%	822	274	Missing	Missing	Missing	274	274	32	220	80.3%	AB665726.1	*Protobothrops flavoviridis*	APKMCGVTETNWKSDEPMKASQILVTPEQQRYLYAPKY	Frame 1
comp78_c0_seq1	AB984590	Metalloprotease P-IIIa 7	1,949	0.2%	489	163	Missing	Missing	Missing	163	163	16	83	50.9%	DQ195153.1	*Viridovipera stejnegeri*	PIKKASQLVVTAEQQRFPRRYVKLAIVADHRMVKKHKE	Frame 1
comp86_c0_seq1	AB984591	Phospholipase B	1,657	0.2%	1,868	622	1-15	16-51	None	553	517	32	274	53.0%	AB848145.1	*Protobothrops flavoviridis*	*ASNLASLSDRLGGLGMIRFGTPSSSDKRPQRCRRWYW	Frame 3
comp102_c0_seq1	AB984592	CRISP	992	0.1%	1,534	511	1-23	24-42	None	240	221	20	154	69.7%	AB848115.1	*Protobothrops flavoviridis*	*LSDFHDFLKATKKLSLHLFKTIEMIAFIVLPILAAVLQQS	Frame 1
comp103_c0_seq1	AB984593	Unknown	1,758	0.2%	474	158	1-3	4-25*	None	42	24	3	24	100.0%	AB440236.1	*Protobothrops flavoviridis*	*RYRMFLCGLGHWVLSLLSFPDPASRITSDQRKRSNLYV	Frame 1
comp87_c0_seq1	AB984594	5'-Nucleotidase	929	0.1%	2,709	903	1-51	52-87	None	588	552	37	318	57.6%	AB848147.1	*Protobothrops flavoviridis*	*RTQPDTSLRLLTRGGIGTAVRLLLPSPSPALLCLKELGG	Frame 3 Reverse
comp81_c0_seq1	AB984595	Nerve Growth Factor	899	0.1%	2,373	791	1-5	None	None	300	295	12	120	40.7%	AB848271.1	*Ovophis okinavensis*	INAEYMGNQYRGGAVLQATSTDCQLPVVQQFDPDWLQ	Frame 3
comp97_c0_seq1	AB984596	Metalloprotease P-III 8	802	0.1%	239	79	Missing	Missing	Missing	79	79	12	62	78.5%	AB074144.1	*Protobothrops flavoviridis*	KLAIVADHGIVTKHHGNLKRIRKWIYQLVNTINNIYRSL	Frame 1
comp132_c0_seq1	AB984597	Phosphodiesterase	561	0.1%	2,700	900	1-39	40-62	None	810	787	67	505	64.2%	AB848153.1	*Protobothrops flavoviridis*	*DWIVPPSLVKNSVRCYPLLLLSQEYPRNPFGSRRNQSG	Frame 2
comp113_c0_seq1	AB984598	C-Type Lectin B Subunit	551	0.1%	735	245	1-35	36-58	None	191	156	10	38	24.4%	GU146049.1	*Protobothrops jerdonii*	STQSTWGGKPGVASEQTCYLWSPRDSSLCREGRKTMG	Frame 1
comp138_c0_seq1	AB984599	C-Type Lectin B Subunit	540	0.1%	608	202	Missing	1-23	None	150	127	17	86	67.7%	AB848139.1	*Protobothrops flavoviridis*	MGRFISVSFGLLVMFLSLSGTGAGFCCPLGWSSYDQHC	Frame 3
comp156_c0_seq1	AB984600	Metalloprotease P-I	539	0.1%	327	109	Missing	Missing	Missing	109	109	0	0	0.0%	DQ464250.1	*Sistrurus catenatus edwardsi*	SHDNAQLLSAIDFNGRTIGLAHVSSMCDPKLSTGIVQDH	Frame 1 Reverse
comp171_c0_seq1	AB984601	C-Type Lectin A Subunit	337	0.0%	1,370	456	Missing	MIssing	None	53	53	6	44	83.0%	AF354924.1	*Viridovipera stejnegeri*	SYREHPLSEGWGGWGASLLLQTSYSWILTLWRELADER	Frame 2
comp268_c0_seq1	AB984602	Galactose-binding Lectin	255	0.0%	589	196	Missing	Missing	None	146	146	6	55	37.7%	AB848130.1	*Protobothrops flavoviridis*	VVFLSLSGAKGSCCPNDSLPMNGMCYKIFDEPKTWED	Frame 1
comp149_c0_seq1	AB984603	QC	240	0.0%	3,804	1,268	1-21	22-54	None	368	335	22	163	48.7%	JF979137.1	*Trimeresurus gracilis*	INAEYMGTHSGGKEGLIGMGISISPAALRRQGKTESWR	Frame 1
comp339_c0_seq1	AB984604	Metalloprotease P-IIId 9	200	0.0%	219	73	Missing	Missing	Missing	72	72	3	44	61.1%	HQ414115.1	*Crotalus adamanteus*	CIALFGPSATVAKDSCFKGNQKGNDYGYCRKENGRMIP	Frame 2
comp425_c0_seq1	AB984605	C-Type Lectin B	197	0.0%	392	130	Missing	1-7	None	130	123	11	94	76.4%	GU146050.1	*Protobothrops jerdonii*	SLSGTGADCPSDWSSYEGHCYRVFKQLKTWEDAEKFC	Frame 3 Reverse
comp507_c0_seq1	AB984606	Metalloprotease P-II 3	174	0.0%	214	71	Missing	Missing	Missing	71	71	9	42	59.2%	AB848137.1	*Protobothrops flavoviridis*	ELGHNLGMRHDGDQCNCTGCIMSAVLSHQPSKLFSNCS	Frame 2 Reverse
comp387_c0_seq1	AB984607	Metalloprotease P-IIa 4	134	0.0%	311	103	Missing	Missing	Missing	103	103	0	0	0.0%	AB851950.1	*Protobothrops flavoviridis*	QLNLTPDEKRFIELVILADHRMFTKYDGDETEIRSRIYES	Frame 3 Reverse
comp190_c0_seq1	AB984608	Serine Protease 7	124	0.0%	1,833	611	Missing	Missing	None	45	45	0	0	0.0%	AB851962.1	*Protobothrops flavoviridis*	*SKIGFFPGVPLNSFERQHFLFQAMRSAPLVGDNGNYSS	Frame 1
comp364_c0_seq1	AB984609	VEGF	99	0.0%	4,636	1,545	1-15	16-40	None	192	166	0	0	0.0%	AB851940.1	*Protobothrops flavoviridis*	*ENPTRAKPPLAPGIMNFLLTWIHWGLAALLYFHNAKV	Frame 1
comp678_c0_seq1	AB984610	Metalloprotease P-II 5	85	0.0%	291	97	Missing	Missing	Missing	96	96	9	56	58.3%	AB848137.1	*Protobothrops flavoviridis*	QLNLTPDQQTYLDAKKYVEFIIVVDHGMVTKYKGDLK	Frame 3 Reverse
comp438_c0_seq1	AB984611	Metalloprotease P-II 6	78	0.0%	345	115	Missing	Missing	Missing	115	115	4	25	21.7%	AY364231.1	*Protobothrops jerdonii*	TETNWESDKPIKKVSQIMIPPEEQRYIELVIVADHRMYTK	Frame 1
comp1053_c0_seq1	AB984612	Metalloprotease P-II 7	51	0.0%	347	115	Missing	Missing	Missing	100	100	0	0	0.0%	AB851942.1	*Protobothrops flavoviridis*	CIALFGPEAEVAPDGCFLNNQKGNDYGYCKKENNTNIP	Frame 3 Reverse
comp997_c0_seq1	AB984613	DPP-IV	42	0.0%	3,504	1,168	1-49	None	None	751	751	0	0	0.0%	AB158224.1	*Gloydius brevicaudus*	*LGAADDPAYPGQEGRVGGREPDERAPAVAVSSLSYRSP	Frame 3 Reverse
comp1091_c0_seq1	AB984614	APA	40	0.0%	3,695	1,231	1-2	None	None	961	959	14	142	14.8%	AB848148.1	*Protobothrops flavoviridis*	*WEMQGMDIEDKSSKMHCMKGKHVAIICGVVIAVGLIL	
