# Supplementary material for: Snake venoms are integrated systems, but abundant venom proteins evolve more rapidly
Source: BMC Genomics. 2015 Aug 28;16:647. doi: 10.1186/s12864-015-1832-6 (PMC4552096; doi:10.1186/s12864-015-1832-6)
Supplement: Supplementary file 3 — Transcript details for the Protobothrops flavoviridis transcriptome. Details as for Additional file 2: Table S1. (PDF 130 kb) [file 12864_2015_1832_MOESM3_ESM.pdf]

ID	DDBJ Accession #	Transcript Toxin Class	FPKM	% FPKM	Transcript Length (bp)	Transcript Length (AA encoded)	5'-UTR (AA)	Signal Peptide (AA)	Prepro-peptide	Transcribed Length (Total AA-5'UTR)	Translated Length (Total AA - (UTR+SP))	# of Unique Peptides Sequenced	# of Amino Acids Sequenced by MS	Peptide Coverage AA%	Nearest BLAST Hit	Nearest Species	Open Reading Frame	Frame
comp40_c0_seq1	AB985221	Phospholipase A_2	128,773	27.6%	810	270	1-12	13-28	None	138	122	34	102	83.6%	AB851917.1	*Protobothrops flavoviridis*	*RGLSIPRSGFGRMRTLWIMAVLLVGVEGGLWQFENMI	Frame 1 Reverse
comp41_c0_seq1	AB985222	Phospholipase A_2	100,786	21.6%	310	103	Missing	Missing	None	86	86	31	84	97.7%	AB072175.1	*Protobothrops flavoviridis*	KDATDRCCFVHDCCYGKVTGCNPKLGKYTYSWNNGD	Frame 2
comp43_c0_seq1	AB985223	Metalloprotease P-IIa 1	39,898	8.6%	2,045	682	1-39	40-59	60-180	483	463	85	321	69.3%	AB052155.1	*Protobothrops flavoviridis*	VSTQSTWGFLASHSQQRKSSDWLESRKRLPVFQPNPAS	Frame 2
comp48_c0_seq1	AB985224	Phospholipase A_2	29,548	6.3%	254	84	Missing	Missing	None	84	84	14	55	65.5%	AB778562.1	*Protobothrops flavoviridis*	DATDRCCFVHDCCYEKLTDCSPKSDIYSYSWKTGVIICG	Frame 2
comp56_c0_seq1	AB985225	LAO	14,483	3.1%	2,865	955	1-22	23-40	None	505	487	109	434	89.1%	AB848142.1	*Protobothrops flavoviridis*	*ALCLASVTFLPSIAIHSLQANKMNVFLMFSLLFLAALG	Frame 3
comp50_c0_seq1	AB985226	Metalloprotease P-II 2	12,698	2.7%	204	68	Missing	Missing	Missing	35	35	0	0	0.0%	AB851953.1	*Protobothrops flavoviridis*	TSADLRKKEQYAGEQGVITRMTAALANLLTVPEIPKQQ	Frame 1
comp46_c0_seq1	AB985227	BPP/CNP	12,302	2.6%	1,615	538	1-76	77-99	None	194	171	1	10	5.8%	AB749764.1	*Protobothrops flavoviridis*	YQRRVHGGERIWPSRQARLDETLLRQSARLDSTRLGSA	Frame 1
comp58_c0_seq1	AB985228	Serine Protease 1	11,386	2.4%	255	85	Missing	Missing	None	50	50	5	46	92.0%	AF159058.1	*Deinagkistrodon acutus*	ICNGQFQGIVSYGGHPCGQSRKPGIYTKVFDYNAWIQSI	Frame 2 Reverse
comp55_c0_seq1	AB985229	C-Type Lectin F IX/X A	10,675	2.3%	742	247	1-31	32-54	None	152	129	30	110	85.3%	AB046491.1	*Protobothrops flavoviridis*	VSTQSTWGKPGVASEQTSYLWRPKEREGRKTMGRFIF	Frame 1
comp49_c0_seq1	AB985230	Serine Protease 2	9,536	2.0%	4,938	1,646	1-28	29-46	None	260	242	50	222	91.7%	AB848162.1	*Protobothrops flavoviridis*	*NILNCAFLPKLFPISKFQANSLLRRVEAMVLIRVLANLL	Frame 1
comp74_c0_seq1	AB985231	Serine Protease 3	9,469	2.0%	216	72	Missing	Missing	None	48	48	5	44	91.7%	AB848158.1	*Protobothrops flavoviridis*	NGQFQGIVHGGGKTCAQPYEPGLYIKVFDYTDWIQNIIA	Frame 1
comp63_c0_seq1	AB985232	CRISP	9,466	2.0%	1,360	453	1-25	26-44	None	240	221	38	176	79.6%	AB848115.1	*Protobothrops flavoviridis*	QRRVHGDDFLKATKKLSLLLFKTIEMIAFIVLPILAAVLQ	Frame 3
comp61_c0_seq1	AB985233	Serine Protease 4	8,139	1.7%	218	73	Missing	Missing	None	54	54	7	50	92.6%	AB848164.1	*Protobothrops flavoviridis*	GGPLICNGEIQGIVSWGGDICAQPHEPGHYTKVFYYIDW	Frame 1
comp53_c0_seq1	AB985234	Serine Protease 5	7,704	1.7%	316	104	Missing	Missing	None	104	104	8	46	44.2%	Q71QI4.1	*Viridovipera stejnegeri*	ECNINEHRFLVALYTFRSRTLHCGGTLINQEWVLSAAHC	Frame 3 Reverse
comp62_c0_seq1	AB985235	VEGF	7,698	1.7%	1,273	424	1-1	2-25	None	146	122	17	98	80.3%	AB848141.1	*Protobothrops flavoviridis*	*AMAAYLLAVAILFCIQGWPSGTVQGQVMPFMEVYSRS	Frame 1
comp69_c0_seq1	AB985236	Metalloprotease P-III 1	7,258	1.6%	203	68	Missing	Missing	None	58	58	11	48	82.8%	AB051849.1	*Protobothrops flavoviridis*	VKCGRLYCIDSSPAKKNPCNIIYSPNDEDKGMVLPGTK	Frame 1
comp64_c0_seq1	AB985237	Serine Protease 6	6,489	1.4%	210	69	Missing	Missing	None	69	69	2	19	27.5%	BAN89399.1	*Protobothrops flavoviridis*	CNINEHRSLALVYITSGFLCGGTLIHPEWVMTAAHCDR	Frame 3
comp65_c0_seq1	AB985238	Metalloprotease P-III 2	5,797	1.2%	563	187	Missing	Missing	Missing	187	187	29	124	66.3%	AB848126.1	*Protobothrops flavoviridis*	EECDCGSPATCRYPCCDAATCKLHSWVECESGECCEQC	Frame 1
comp76_c0_seq1	AB985239	C-Type Lectin F IX/X B	5,458	1.2%	489	163	1-2	3-25	None	146	123	27	114	92.7%	D83332.1	*Protobothrops flavoviridis*	KTMGRFIFVSFGLLVVFLSLSGTAADCPSDWSSYEGHCY	Frame 1
comp72_c0_seq1	AB985240	Metalloprotease P-III 3	3,351	0.7%	353	117	Missing	Missing	Missing	117	117	7	75	64.1%	AB051849.1	*Protobothrops flavoviridis*	AEGLCCDQCRFKGAGTECRAATDECDMADLCTGRSAE	Frame 2 Reverse
comp75_c0_seq1	AB985241	Metalloprotease P-III 4	3,169	0.7%	754	250	Missing	Missing	Missing	250	250	57	189	75.6%	AB848128.1	*Protobothrops flavoviridis*	TNWESDEPIKKASKLVVTAEQQRYLNNFRFIELVIVADY	Frame 3 Reverse
comp78_c0_seq1	AB985242	Metalloprotease P-III 5	2,853	0.6%	453	151	Missing	Missing	Missing	136	136	13	86	63.2%	AB665726.1	*Protobothrops flavoviridis*	HRNGQPCLNNHGYCYNGNCPIMLHQCITLFGLGATVD	Frame 1
comp101_c0_seq1	AB985243	C-Type Lectin A	2,702	0.6%	412	137	Missing	Missing	None	127	127	26	127	100.0%	AB848117.1	*Protobothrops flavoviridis*	SAYDRYCYQAFSKPKNWEDAESFCEEGVKTSHLVSIESS	Frame 1
comp91_c0_seq1	AB985244	Metalloprotease P-III 6	2,148	0.5%	756	252	Missing	Missing	Missing	252	252	40	206	81.7%	AB848138.1	*Protobothrops flavoviridis*	WESDESIKEDSQSNLTPAQQKYLNAKKYVKFFLVADHI	Frame 1 Reverse
comp93_c0_seq1	AB985245	Serine Protease 7	1,972	0.4%	215	71	Missing	Missing	None	71	71	3	9	12.7%	AB851963.1	*Protobothrops flavoviridis*	KLRGAMCVLLLTELSSLINMMSLSTSSSFFLLGQKNFSF	Frame 3
comp139_c0_seq1	AB985246	C-Type Lectin B	1,594	0.3%	344	113	Missing	Missing	None	113	113	39	113	100.0%	AB848140.1	*Protobothrops flavoviridis*	EHCYQVFQQKMNWADAEKFCTQQHKGSHLVSFHSSEE	Frame 2 Reverse
comp86_c0_seq1	AB985247	5'-Nucleotidase	1,529	0.3%	2,581	860	1-51	None	None	591	591	60	382	64.6%	AB848147.1	*Protobothrops flavoviridis*	*RTQPDTSLRLLTRGGIGTAVRLLLPSPSPALLCLKELGG	Frame 1
comp125_c0_seq1	AB985248	Phospholipase B	1,228	0.3%	1,861	620	1-15	16-51	None	553	517	30	248	48.0%	AB848155.1	*Protobothrops flavoviridis*	*ASNLAPLSDRLGGLGMIRFGTPSSSDKRRQRCRSWYW	Frame 1
comp173_c0_seq1	AB985249	Metalloprotease P-IIIb 7	1,138	0.2%	257	85	Missing	Missing	Missing	85	85	0	0	0.0%	AB074144.1	*Protobothrops flavoviridis*	HRNGQPCLYNHGYCYNGKCPIMFYQCYFLFGSNATVA	Frame 1
comp157_c0_seq1	AB985250	Serine Protease 8	1,065	0.2%	207	68	Missing	Missing	None	68	68	1	12	17.6%	AB848157.1	*Protobothrops flavoviridis*	LTAAHCDRSSIYMYIGMHDENVKFDDEQGRSPKEKYFY	Frame 1
comp141_c0_seq1	AB985251	Metalloprotease P-III 8	1,023	0.2%	264	87	Missing	Missing	Missing	87	87	6	61	70.1%	AB848156.1	*Protobothrops flavoviridis*	WRETVLLNRKNHDNTQLLTGIDFDGNTIGFGYIGSMCT	Frame 2 Reverse
comp115_c0_seq1	AB985252	Phosphodiesterase	894	0.2%	2,825	942	1-39	40-62	None	851	828	78	532	64.3%	AB848150.1	*Protobothrops flavoviridis*	*DRIVPPSLVKNSVCCYPLPLLSQEYPRNPFGSRRNQSG	Frame 3
comp189_c0_seq1	AB985253	C-Type Lectin F IX/X A	694	0.1%	210	70	Missing	Missing	None	47	47	0	0	0.0%	AB848120.1	*Protobothrops flavoviridis*	SSVSYENLVKPFSKKCFVLKKESEFRKWFNVYCGQQY	Frame 3
comp204_c0_seq1	AB985254	Metalloprotease P-III 9	693	0.1%	201	67	Missing	Missing	Missing	67	67	0	0	0.0%	AB074144.1	*Protobothrops flavoviridis*	VAVIMTHEMGHNLGIPHDGNSCTCGGFPCIMSPMISDPP	Frame 1 Reverse
comp166_c0_seq1	AB985255	Metalloprotease P-III 10	664	0.1%	315	104	Missing	Missing	Missing	104	104	8	45	43.3%	AB848114.1	*Protobothrops flavoviridis*	NWESDEPMKASQILLTPEQQRYLYAPKYIKLVIVADDV	Frame 2 Reverse
comp85_c0_seq1	AB985256	Nerve Growth Factor	459	0.1%	4,805	1,602	1-59	60-77	None	241	223	4	45	20.2%	AB848144.1	*Protobothrops flavoviridis*	INAEYMGNQYRGGAVLQATSTDCQLPVVQQFDPDWLQ	Frame 2 Reverse
comp196_c0_seq1	AB985257	QC	375	0.1%	3,643	1,214	1-88	89-111	None	368	345	18	170	49.3%	AB848133.1	*Protobothrops flavoviridis*	*LGWELASLPPRYVGKARQKAGREAKSASGRQKRSRP	Frame 2 Reverse
comp20_c0_seq1	AB985258	VEGF	310	0.1%	4,350	1,450	1-70	71-96	None	192	166	2	58	34.9%	AB851940.1	*Protobothrops flavoviridis*	*AEDPERAAKRGQTTSLGPRESRAYGRVKPESNILPPLP	Frame 2
comp268_c0_seq1	AB985259	Metalloprotease P-III 11	239	0.1%	280	92	Missing	Missing	Missing	92	92	1	12	13.0%	AB074144.1	*Protobothrops flavoviridis*	VTAEQQRFPRRYIKLAIVVDHGIVTKHHGNLKKIRKWIY	Frame 3
comp595_c0_seq1	AB985260	Metalloprotease P-II 3	114	0.0%	518	172	Missing	Missing	Missing	172	172	10	79	45.9%	AB848137.1	*Protobothrops flavoviridis*	VTETNWESYEPIKKAFQLNLTPKQQRYLDAKKYVEFIV	Frame 1
comp400_c0_seq1	AB985261	Hyaluronidase	113	0.0%	1,798	599	1-77	None	None	526	526	0	0	0.0%	AB851937.1	*Protobothrops flavoviridis*	INAEYMGIWKVVLLILFWTSSFSGGLRLVFASLGLWNY	Frame 1
comp566_c0_seq1	AB985262	Metalloprotease P-IIa 4	105	0.0%	276	92	Missing	Missing	Missing	92	92	3	32	34.8%	AB851950.1	*Protobothrops flavoviridis*	IELVIVADHRMFTKYEGDETEIHSRIYESVNALNVIFRAL	Frame 1
comp1106_c0_seq1	AB985263	DPP IV	52	0.0%	2,812	937	1-49	None	None	751	751	0	0	0.0%	AB851922.1	*Protobothrops flavoviridis*	*LGAADDPAYPGQEGRVGGREPDERAPAVAVSSLSHRFP	Frame 3 Reverse
comp1656_c0_seq1	AB985264	APA	30	0.0%	3,710	1,237	1-5	None	None	955	955	9	89	9.3%	AB848148.1	*Protobothrops flavoviridis*	*WEMQGMDIEDKSSKMHCMKGKHVAIICGVVIAVGLIL	Frame 2
comp552_c0_seq1	AB985265	Phospholipase A_2	14	0.0%	1,258	419	Missing	Missing	None	81	81	1	7	8.6%	Q2PG81.2	*Protobothrops elegans*	DRCCFVHRCCYKKVTGCDPKKDRYSYSWENKAIVCGE	Frame 3
comp87_c0_seq1	AB985266	C-Type Lectin B	15	0.0%	464	155	Missing	1-23	None	150	127	25	105	82.7%	AB848139.1	*Protobothrops flavoviridis*	MGRFIFVRFSLLVVAVSLSGTGAGFCCPLGWSSYDQHC	Frame 2
