# Supplementary material for: Snake venoms are integrated systems, but abundant venom proteins evolve more rapidly
Source: BMC Genomics. 2015 Aug 28;16:647. doi: 10.1186/s12864-015-1832-6 (PMC4552096; doi:10.1186/s12864-015-1832-6)
Supplement: Supplementary file 4 — Phospholipases A2 in venoms of P. elegans, P. flavoviridis, and hybrids between the two. A. Myotoxic PLA2s. Catalytic PLA2s have a critical Asp residue in position 48 (See panels B-D). All of these enzymes manifest a basic residue in this position, preventing the binding of a critical Ca2+ ion involved in catalysis. However, while most New World crotaline myotoxins have Lys at this position, P. elegans and some other Asian crotalines have substituted Arg. Partial transcript comp552_c0_seq1 from P. flavoviridis is also a myotoxin, but it is a minor venom component (Additional file 3: Table S2). Signal peptides are shown to the left of the vertical black line. B. Acidic PLA2s. These enzymes are strongly lipolytic and myotoxic, though much less myotoxic than the non-catalytic myotoxins shown in panel D. C. Moderately basic PLA2s. P. flavoviridis PLA2 1 is most similar to PL-Y, PL-X, and PLA-B. Like those in panel B, these enzymes are strongly lipolytic and moderately myotoxic. PLA-B induces edema [12]. D. Weakly neurotoxic PLA2s. The partial transcript, P. flavoviridis comp48_c0_seq1 is identical, as far as can be seen to PLA-N(O) [11]. (PDF 140 kb) [file 12864_2015_1832_MOESM4_ESM.pdf]

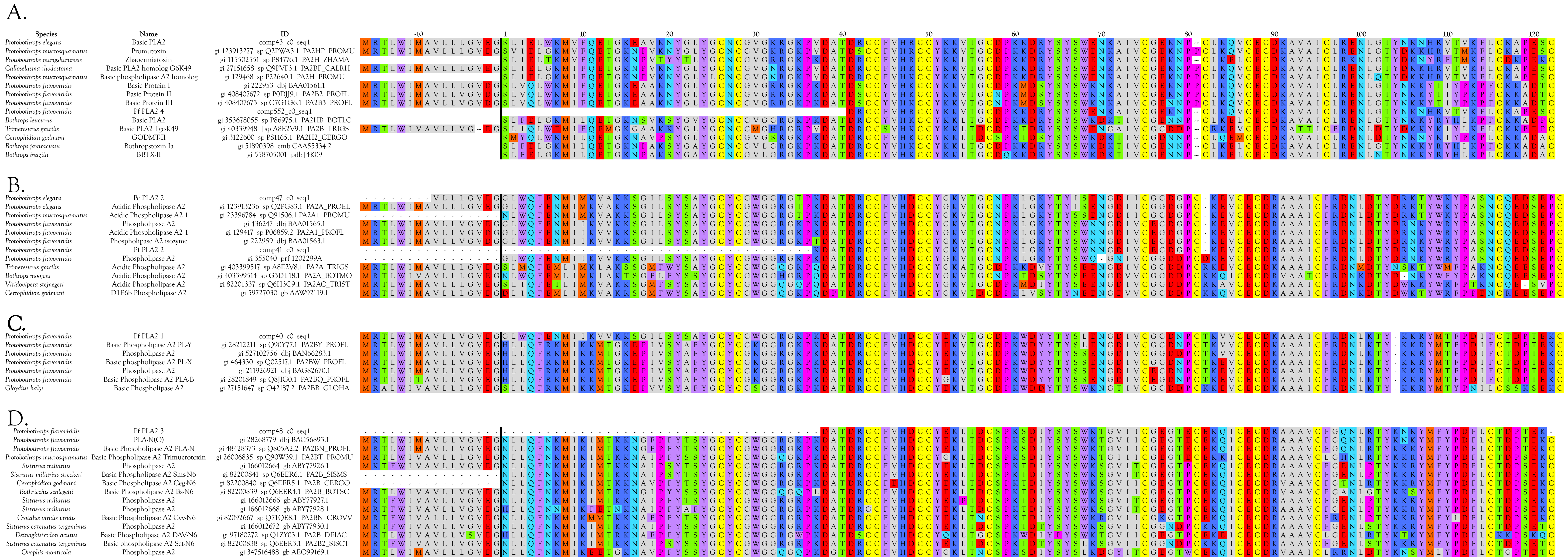
A.
Species
Name
ID
B.
C.
D.
