# Supplementary figures and images for: Snake venoms are integrated systems, but abundant venom proteins evolve more rapidly
Source: BMC Genomics. 2015 Aug 28;16:647. doi: 10.1186/s12864-015-1832-6 (PMC4552096; doi:10.1186/s12864-015-1832-6)

Pe\_comp43\_c0\_seq1

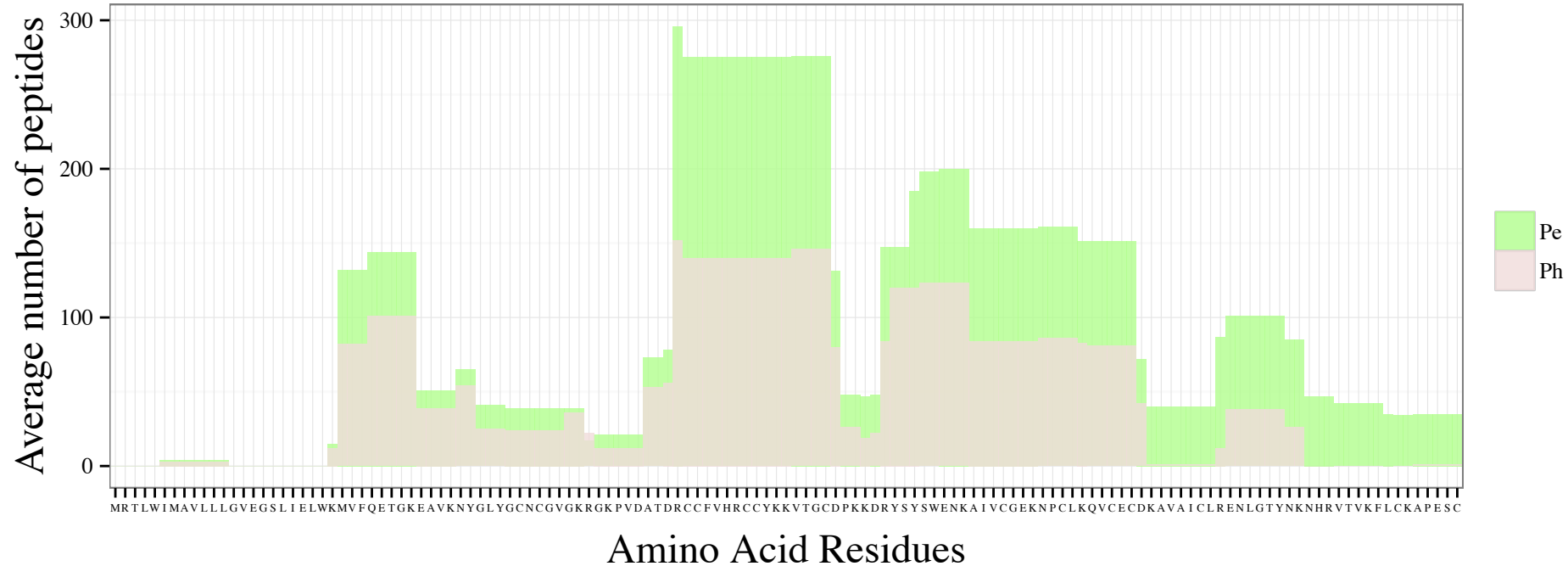

Supplement: Supplementary file 5 — Mean frequency per specimen with which individual amino acid residues in the noncatalytic, myotoxic P. elegans PLA2, comp43_c0_seq1, were sequenced in P. elegans and hybrid venoms. Not only were most of the same peptides sequenced in these venoms, but they were sequenced with the same relative frequencies. (PDF 234 kb) [file 12864_2015_1832_MOESM5_ESM.pdf]

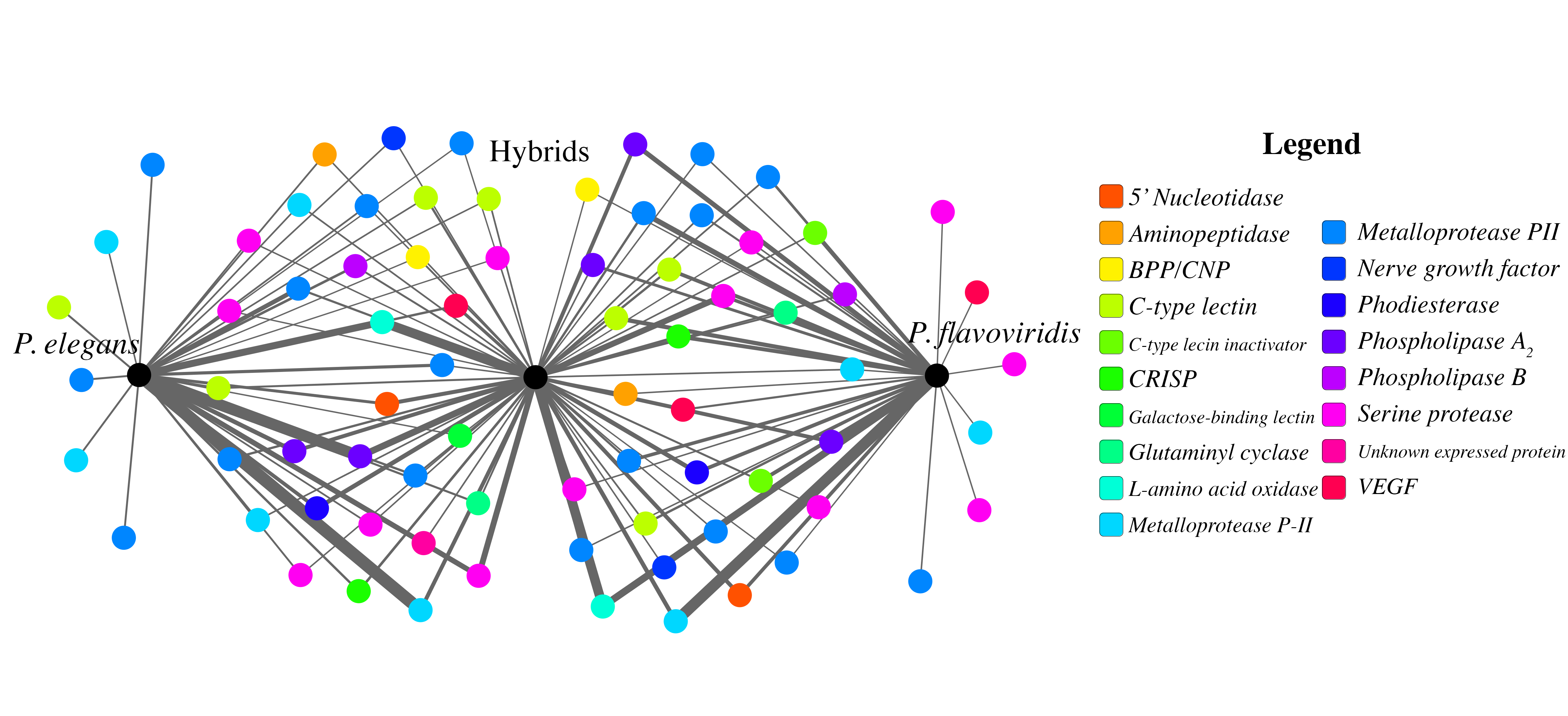

Supplement: Supplementary file 6 — Hybrids express most proteins found in the two parental species. Every colored node corresponds to a transcript and is color-coded by toxin family. The toxins are connected to black nodes labeled with species identities, by edges whose thickness corresponds to the protein expression level in that species. Expression values are given as a percentage of the total, averaged across all samples, and range from less than 0.001 to 19 %. More detail on expression levels of individual toxins is presented in Fig 3. Virtually all toxins are expressed in the hybrids, except for a few venom components, which are poorly expressed in the parental species, and may have been missed in the hybrids due stochasticity involved in mass spectrometric detection. (PNG 1018 kb) [file 12864_2015_1832_MOESM6_ESM.png]

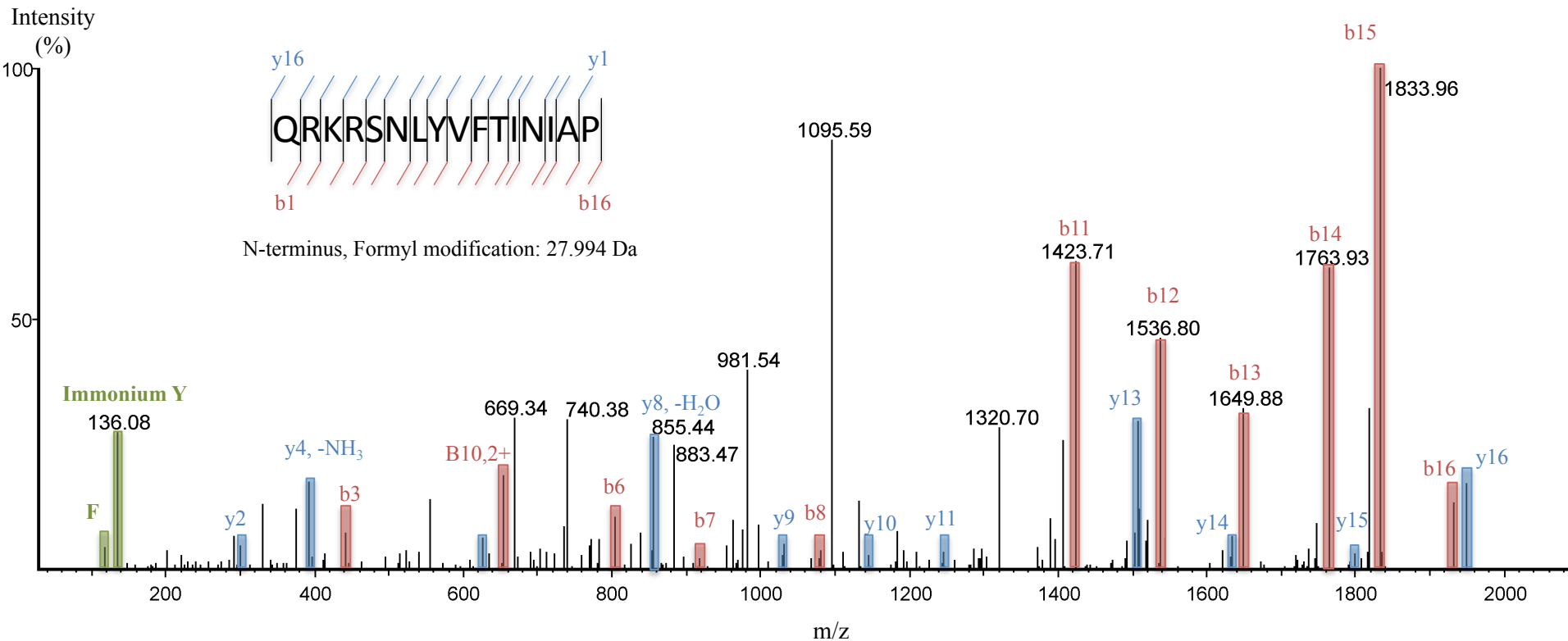

Supplement: Supplementary file 7 — Manual identification of CID fragment ions from a composite spectrum using >40 MS2 spectra from the same chromatographic peak associated with Protobothrops elegans transcript comp103_c0_seq1. Annotation was performed using the PEAKS studio 7.0 de novo sequencing module, followed by a search with the same database used for Mascot searches, and manual annotation to ascertain correctness. This process confirmed the sequence of the peptide and that Frame 1 is correct frame for translation. (PDF 320 kb) [file 12864_2015_1832_MOESM7_ESM.pdf]

Residue Abundance/Specimen

Pe\_comp47\_c0\_seq1

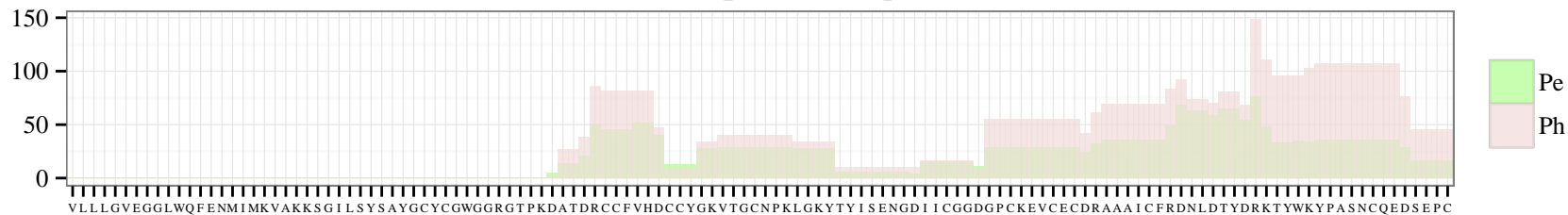

Pf\_comp41\_c0\_seq1

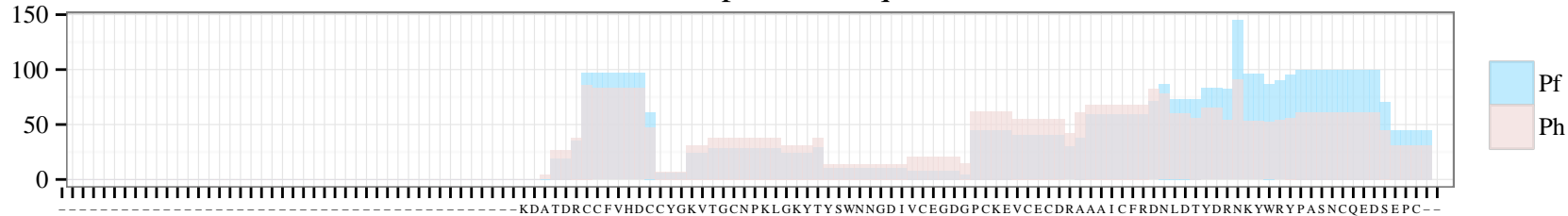

Amino Acid Residues

Supplement: Supplementary file 8 — Venom peptides sequenced from both parental taxa and hybrids pertaining to transcripts for the homologous, basic, catalytic PLA2s, P. elegans comp47_c0_seq1 and P. flavoviridis comp41_c0_seq1. Hybrids inherited both homologs. (PDF 12 kb) [file 12864_2015_1832_MOESM8_ESM.pdf]
