# Supplementary material for: Snake venoms are integrated systems, but abundant venom proteins evolve more rapidly
Source: BMC Genomics. 2015 Aug 28;16:647. doi: 10.1186/s12864-015-1832-6 (PMC4552096; doi:10.1186/s12864-015-1832-6)
Supplement: Supplementary file 9 — Supplementary Material. (DOCX 70 kb) [file 12864_2015_1832_MOESM9_ESM.docx]

# Supplementary Material

## Phospholipases A_2_.

The *P. elegans* myotoxic PLA_2_ possesses an arginine residue at position 48, where catalytic PLA_2_s have an aspartic acid residue (Figure S1B-D), and where most other Asian and all American crotaline PLA_2_ myotoxins have lysine (Figure S1A; Table S1). This Lys-Arg mutation appears to have occurred within the Genus *Protobothrops*. Interestingly, the *Protobothrops* myotoxic PLA_2_s do not appear a good fit for any of the four best models for this PLA_2_ subclass [1-4], nicely summarized by Lomonte and Rangel [5] (Figure S1A). The Selistre de Araujo et al. model, which emphasizes the importance of N-terminal residues, appears to provide the best fit for the Asian crotaline (*Protobothrops*, *Calloselasma*, *Trimeresurus*) enzymes, which have the requisite K7, E12, T13, and K15 (16 in Selistre de Araujo et al.). Some sequences have N16 (17), but many have E16 or even G16. K78 is present in some sequences, but in fact, a BLAST search for the top 100 myotoxic PLA_2_s most closely related to *P. elegans* comp43_c0_seq1 located only 16 toxins with K78. Most others have N, D, or S, and several have either E or G. K117 (116) is commonly present, but K118 (117) is often replaced by A. The Chioato et al. model stressed the importance of C-terminal residues, most of which appear to be absent in the Asian enzymes. The dos Santos model focuses on the importance of K20, K115, R118, and Y119. The Asian enzymes have K19 (zhaoermiatoxin has T19), K113, and K117, but the Arg and Tyr residues do not exist (Figure S1A).

There have been fewer attempts to unravel the structural determinants of PLA_2_ neurotoxicity than of myotoxicity [6, 7]. Tsai and Wang [8] used site-directed mutagenesis to probe the role of N6 (Figure S1D) in neurotoxicity of trimucrotoxin. This asparagine residue is almost invariant among Old World crotaline neurotoxic PLA_2_s and is common to many New World homologs as well. N6A and N6E mutants retained more than half their phospholipolytic activity, but lost 67% and 90% of their neurotoxicity, respectively. Sribar et al. [9, 10] identified calmodulin and 14-3-3 protein as targets of the viperid ammodytoxin C; however, given the structural diversity of presynaptic PLA_2_s, it may be that different presynaptic neurotoxins have different protein targets on nerve termini and that they interact with those targets via different structural features [7]. And once again there is the question of prey chemistry. Different vertebrate prey organisms may be targeted by different PLA_2_ chemistries.

The first two *P. flavoviridis* transcripts are similar to PL-Y [11] from the same venom. The most abundant of these differs from PL-Y because of three frame-shift mutations in the N-terminal 23 residues. PL-Y does not promote edema, but beyond this, there is no indication of its pharmacological function. PLA-B, in the same group, is inflammatory and induces edema [12]. The third *P. flavoviridis* PLA_2_ transcript is catalytic and is identical to PLA-N (O), as far as our partial transcript will allow us to compare [11, 13]. PLA-N (O) is weakly neurotoxic (Figure S1D; Table S2). In addition to its neurotoxicity, this PLA_2_ is strongly cytotoxic to HL-60 cancer cells (Oda-Ueda, in [11]).

## Pharmacology of thrombin-like serine proteases relative to envenomation.

In normal blood clotting, damaged vascular epithelium releases tissue plasminogen activator (tPA), which is complexed with plasminogen activator inhibitor-1 [14]. tPA activates plasminogen trapped in the clots, resulting in their degradation. Hemorrhagic metalloproteases, which are so abundant in many pit viper venoms, probably also trigger tPA release, although this would be slower than direct plasmin activation. The latter is accomplished by venom plasminogen activators that convert plasminogen to plasmin to hydrolyze fibrin into peptides that are cleared by both endogenous and exogenous proteases and peptidases. Regardless of the effect of hemorrhagins, Sunagawa et al. [15] have shown that 50 nM habutobin, a TLE from the venom of *P. flavoviridis*, causes a significant release of tPA and urokinase-type PA from cultured bovine pulmonary artery endothelial cells.

Many snake venom TLEs clot fibrinogen less effectively than thrombin [16]. For instance, grambin, from *Trimeresurus gramineus* venom, preferentially removes fibrinopeptide A from fibrinogen, but releases only trace quantities of fibrinopeptide B [17]. Another TLE from venom of *Gloydius halys* released fibrinopeptide B first, followed slowly by fibrinopeptide A, and it clots fibrinogen very weakly [18-20]. TLEs are more effective against fibrinogens of some mammal species than others [21], but the existence of so many weakly clotting TLEs, the capacity of various crotaline TLEs to degrade prothrombin [22], and the existence of directly fibrinolytic venom enzymes, suggest that the objective is not to clot blood, but to clear the bloodstream of fibrinogen [23]. In addition to hydrolyzing fibrin, plasmin also inactivates many endogenous clotting factors, thereby acting as an anticoagulant [24]; however, this also suggests that the strategy may be to prevent endogenous coagulation factors from producing properly clotted fibrin.

Snake venoms are redundant systems, often employing multiple lines of attack on the same pharmacological target (e.g. dendrotoxins, fasciculins, and acetylcholine in mamba venoms) [25]. In addition to activating thrombin, cleaving fibrinogen directly, and activating plasmin, snake venoms are also capable of inactivating human serine protease inhibitors (serpins). Kress [26] reported that antithrombin III, C-1 inhibitor, α_1_-antitrypsin inhibitor, α_2_-antiplasmin, and α_1_-anti-chymotrypsin inhibitor were inactivated by proteases from venoms of *Crotalus atrox* and *Crotalus adamanteus*. Urano et al. [27] found that the thrombin-like enzyme, reptilase, was able to directly inactivate human plasminogen activator inhibitor-1 and α_2_-antiplasmin, but that two other venom TLEs were unable to do so. Other metallo- and serine proteases directly digest fibrin or activate Protein C [28-30].

## Evolutionary rate data.

The evolutionary rate data are extremely interesting, but their interpretation at the level of individual protein classes is entirely speculative at this point, given the stochasticity associated with protein evolution. However, some observations merit further consideration, and for that reason, we have offered them here.

The high dN/dS values of PLA_2_s, P-II MPs, and CTLs are not surprising, given the tremendous diversity of those toxin families (Table S4). The low dN/dS values for serine proteases and P-III MPs are surprising, but we suspect that this reflects the number of incomplete transcripts, and the degree of incompleteness of many of them. Glutaminyl cyclase showed the lowest dN/dS ratio of all (Table S4), but it is not strictly a venom protein. The function of this enzyme is to cyclize the N-terminal glutamine residues of various venom proteins (e.g. acidic subunit of crotoxin, BPPs, etc.). It functions in the gland and has no known function in the prey.

5’-nucleotidase is a venom enzyme, but the few published sequences show very little primary structural variation. How does one explain this? Kini and colleagues [31, 32] have argued persuasively that surface residues on toxins are involved in targeting the toxins to specific prey proteins. 5’-nucleotidase is an exception. It hydrolyzes 5’-mononucleotides, which are structurally invariant in all vertebrates and invertebrates. While the abundance of 5’-nucleotidase could be modulated strategically, 5’-mononucleotide concentrations probably do not vary excessively in different vertebrate tissues, which may explain the low levels of this enzyme detected in most venoms to date. Venom phosphodiesterase (PDE), which is biochemically and strategically linked to 5’-nucleotidase shows a slightly higher dN/dS ratio. PDEs, as a protein family, are more diverse than the latter, hydrolyzing a greater array of oligonucleotide substrates.

L-amino acid oxidase also has a relatively low dN/dS ratio. LAO oxidizes amino acids to liberate H_2_O_2_, by which it inhibits platelet aggregation [33, 34] and activates soluble guanylate cyclase to promote hypotension. LAO prefers aromatic and hydrophobic amino acids as substrates [35, 36]. Snake venom leucine aminopeptidase (LAP) [37], ecto-LAP [38], and venom hemorrhagic MPs preferentially release LAO’s preferred amino acids [39-46]. Again, however, LAO does not need to interact with prey proteins. It simply needs to oxidize a relatively small number of amino acids, the structures of which do not vary among prey.

# Supplementary Literature Cited

1. Lomonte B, Moreno E, Tarkowski A, Hanson LA, Maccarana M: **Neutralizing interaction between heparins and myotoxin II, a lysine 49 phospholipase A2 from Bothrops asper snake venom. Identification of a heparin-binding and cytolytic toxin region by the use of synthetic peptides and molecular modeling**. *J Biol Chem* 1994, **269**(47):29867-29873.

2. Selistre de Araujo HS, White SP, Ownby CL: **cDNA cloning and sequence analysis of a lysine-49 phospholipase A2 myotoxin from Agkistrodon contortrix laticinctus snake venom**. *Arch Biochem Biophys* 1996, **326**(1):21-30.

3. Chioato L, De Oliveira AH, Ruller R, Sa JM, Ward RJ: **Distinct sites for myotoxic and membrane-damaging activities in the C-terminal region of a Lys49-phospholipase A2**. *Biochem J* 2002, **366**(Pt 3):971-976.

4. dos Santos JI, Fernandes CA, Magro AJ, Fontes MR: **The intriguing phospholipases A2 homologues: relevant structural features on myotoxicity and catalytic inactivity**. *Protein Pept Lett* 2009, **16**(8):887-893.

5. Lomonte B, Rangel J: **Snake venom Lys49 myotoxins: From phospholipases A(2) to non-enzymatic membrane disruptors**. *Toxicon* 2012, **60**(4):520-530.

6. Kini RM, Iwanaga S: **Structure-function relationships of phospholipases. I: Prediction of presynaptic neurotoxicity**. *Toxicon* 1986, **24**(6):527-541.

7. Prijatelj P, Jenko Praznikar Z, Petan T, Krizaj I, Pungercar J: **Mapping the structural determinants of presynaptic neurotoxicity of snake venom phospholipases A2**. *Toxicon* 2008, **51**(8):1520-1529.

8. Tsai IH, Wang YM: **Effect of site directed mutagenesis on the activity of recombinant trimucrotoxin, a neurotoxic phospholipase from Trimeresurus mucrosquamatus venom**. *Toxicon* 1998, **36**(11):1591-1597.

9. Sribar J, Copic A, Paris A, Sherman NE, Gubensek F, Fox JW, Krizaj I: **A high affinity acceptor for phospholipase A2 with neurotoxic activity is a calmodulin**. *J Biol Chem* 2001, **276**(16):12493-12496.

10. Sribar J, Copic A, Poljsak-Prijatelj M, Kuret J, Logonder U, Gubensek F, Krizaj I: **R25 is an intracellular membrane receptor for a snake venom secretory phospholipase A(2)**. *FEBS Lett* 2003, **553**(3):309-314.

11. Chijiwa T, Yamaguchi Y, Ogawa T, Deshimaru M, Nobuhisa I, Nakashima K, Oda-Ueda N, Fukumaki Y, Hattori S, Ohno M: **Interisland evolution of *Trimeresurus flavoviridis* venom phospholipase A_2_ isozymes**. *Journal of molecular evolution* 2003, **56**(3):286-293.

12. Yamaguchi Y, Shimohigashi Y, Chijiwa T, Nakai M, Ogawa T, Hattori S, Ohno M: **Characterization, amino acid sequence and evolution of edema-inducing, basic phospholipase A2 from Trimeresurus flavoviridis venom**. *Toxicon* 2001, **39**(7):1069-1076.

13. Ikeda N, Chijiwa T, Matsubara K, Oda-Ueda N, Hattori S, Matsuda Y, Ohno M: **Unique structural characteristics and evolution of a cluster of venom phospholipase A(2) isozyme genes of Protobothrops flavoviridis snake**. *Gene* 2010.

14. Schreiber SS, Tan Z, Sun N, Wang L, Zlokovic BV: **Immunohistochemical localization of tissue plasminogen activator in vascular endothelium of stroke-prone regions of the rat brain**. *Neurosurgery* 1998, **43**(4):909-913.

15. Sunagawa M, Hanashiro K, Nakamura M, Kosugi T: **Habutobin releases plasminogen activator (U-PA) from bovine pulmonary artery endothelial cells**. *Toxicon* 1996, **34**(6):691-699.

16. Wei WL, Sun JJ, Chen JS: **Synergism of procoagulation effect of thrombin-like enzymes from Deinagkistrodon acutus and Agkistrodon halys snake venoms**. *Zhongguo Yao Li Xue Bao* 1996, **17**(6):527-531.

17. Chang MC, Huang TF: **Characterization of a thrombin-like enzyme, grambin, from the venom of Trimeresurus gramineus and its in vivo antithrombotic effect**. *Toxicon* 1995, **33**(8):1087-1098.

18. Shu YY, Moran JB, Geren CR: **A thrombin-like enzyme from timber rattlesnake venom**. *Biochim Biophys Acta* 1983, **748**(2):236-244.

19. Guan LF, Chi CW, Yuan M: **Study on the thrombin-like enzyme preferentially releasing fibrinopeptide B from the snake venom of Agkistrodon halys Pallas**. *Thromb Res* 1984, **35**(3):301-310.

20. Jin Y, Lu QM, Chen RQ, Wu JB, Xiong YL: **Molecular characterization of a weak fibrinogen-clotting enzyme from Trimeresurus jerdonii venom**. *Toxicon* 2005, **45**(3):353-360.

21. Santoro ML, Sano-Martins IS: **Different clotting mechanisms of Bothrops jararaca snake venom on human and rabbit plasmas**. *Toxicon* 1993, **31**(6):733-742.

22. Pirkle H, Markland FS, Theodor I: **Thrombin-like enzymes of snake venoms: actions on prothrombin**. *Thromb Res* 1976, **8**(5):619-627.

23. Swenson S, Markland FS, Jr.: **Snake venom fibrin(ogen)olytic enzymes**. *Toxicon* 2005, **45**(8):1021-1039.

24. Hoover-Plow J: **Does plasmin have anticoagulant activity?** *Vascular health and risk management* 2010, **6**:199-205.

25. Aird SD: **Ophidian envenomation strategies and the role of purines.** *Toxicon* 2002, **40**(4):335-393.

26. Kress LF: **Inactivation of human plasma serine proteinase inhibitors (serpins) by limited proteolysis of the reactive site loop with snake venom and bacterial metalloproteinases**. *J Cell Biochem* 1986, **32**(1):51-58.

27. Urano T, Ihara H, Takada Y, Fujie M, Takada A: **The cleavage and inactivation of plasminogen activator inhibitor type 1 and alpha2-antiplasmin by reptilase, a thrombin-like venom enzyme**. *Blood Coagul Fibrinolysis* 2000, **11**(2):145-153.

28. Retzios AD, Markland FS, Jr.: **A direct-acting fibrinolytic enzyme from the venom of Agkistrodon contortrix contortrix: effects on various components of the human blood coagulation and fibrinolysis systems**. *Thromb Res* 1988, **52**(6):541-552.

29. Randolph A, Chamberlain SH, Chu HL, Retzios AD, Markland FS, Jr., Masiarz FR: **Amino acid sequence of fibrolase, a direct-acting fibrinolytic enzyme from Agkistrodon contortrix contortrix venom**. *Protein Sci* 1992, **1**(5):590-600.

30. Stocker K, Fischer H, Meier J, Brogli M, Svendsen L: **Protein C activators in snake venoms**. *Behring Inst Mitt* 1986(79):37-47.

31. Kini RM, Evans HJ: **A model to explain the pharmacological effects of snake venom phospholipases A2**. *Toxicon* 1989, **27**(6):613-635.

32. Kini RM, Chan YM: **Accelerated evolution and molecular surface of venom phospholipase A2 enzymes**. *Journal of molecular evolution* 1999, **48**(2):125-132.

33. Nathan I, Dvilansky A, Yirmiyahu T, Aharon M, Livne A: **Impairment of platelet aggregation by Echis colorata venom mediated by L-amino acid oxidase or H2O2**. *Thromb Haemost* 1982, **48**(3):277-282.

34. Takatsuka H, Sakurai Y, Yoshioka A, Kokubo T, Usami Y, Suzuki M, Matsui T, Titani K, Yagi H, Matsumoto M *et al*: **Molecular characterization of L-amino acid oxidase from Agkistrodon halys blomhoffii with special reference to platelet aggregation**. *Biochim Biophys Acta* 2001, **1544**(1-2):267-277.

35. Tan NH, Swaminathan S: **Purification and properties of the L-amino acid oxidase from monocellate cobra (Naja naja kaouthia) venom**. *Int J Biochem* 1992, **24**(6):967-973.

36. Pessatti M, Fontana JD, Furtado MF, Guimaraes MF, Zanette LR, Costa WT, Baron M: **Screening of Bothrops snake venoms for L-amino acid oxidase activity**. *Appl Biochem Biotechnol* 1995, **51-52**:197-210.

37. Tu AT, Toom PM: **The presence of a L-leucyl-beta-napthylamide hydrolyzing enzyme in snake venoms**. *Experientia* 1967, **23**(6):439-440.

38. Nagaoka I, Yamashita T: **Inactivation during phagocytosis of leucine aminopeptidase, an ecto-enzyme of polymorphonuclear neutrophils**. *Biochim Biophys Acta* 1981, **678**(3):342-351.

39. Nikai T, Mori N, Kishida M, Sugihara H, Tu AT: **Isolation and biochemical characterization of hemorrhagic toxin f from the venom of *Crotalus atrox* (Western diamondback rattlesnake)**. *Arch Biochem Biophys* 1984, **231**(2):309-319.

40. Kishida M, Nikai T, Mori N, Kohmura S, Sugihara H: **Characterization of mucrotoxin A from the venom of Trimeresurus mucrosquamatus (the Chinese habu snake)**. *Toxicon* 1985, **23**(4):637-645.

41. Hagihara S, Komori Y, Tu AT: **Proteolytic specificity of hemorrhagic toxin b from *Crotalus atrox* (Western diamondback rattlesnake) venom**. *Comp Biochem Physiol* 1985, **82C**(1):21-27.

42. Bjarnason JB, Hamilton D, Fox JW: **Studies on the mechanism of hemorrhage production by free proteolytic hemorrhagic toxin from *Crotalus atrox* venom.** *Biol Chem Hoppe-Seyler* 1988, **369**(Suppl.):121-129.

43. Baramova EN, Shannon JD, Fox JW, Bjarnason JB: **Proteolytic digestion of non-collagenous basement membrane proteins by the hemorrhagic metalloproteinase Ht-e from Crotalus atrox venom**. *Biomed Biochim Acta* 1991, **50**(4-6):763-768.

44. Guan AL, Retzios AD, Henderson GN, Markland FS, Jr.: **Purification and characterization of a fibrinolytic enzyme from venom of the southern copperhead snake (Agkistrodon contortrix contortrix)**. *Arch Biochem Biophys* 1991, **289**(2):197-207.

45. Sanchez EF, Cordeiro MN, De Oliveira EB, Juliano L, Prado ES, Diniz CR: **Proteolytic specificity of two hemorrhagic factors, LHF-I and LHF-II, isolated from the venom of the bushmaster snake (Lachesis muta muta)**. *Toxicon* 1995, **33**(8):1061-1069.

46. Kurtovic T, Brgles M, Leonardi A, Balija ML, Krizaj I, Allmaier G, Marchetti-Deschmann M, Halassy B: **Ammodytagin, a heterodimeric metalloproteinase from Vipera ammodytes ammodytes venom with strong hemorrhagic activity**. *Toxicon* 2011, **58**(6-7):570-582.
